# How Food Controls Aggression in *Drosophila*

**DOI:** 10.1371/journal.pone.0105626

**Published:** 2014-08-27

**Authors:** Rod S. Lim, Eyrún Eyjólfsdóttir, Euncheol Shin, Pietro Perona, David J. Anderson

**Affiliations:** 1 Division of Biology and Biological Engineering, California Institute of Technology, Pasadena, California, United States of America; 2 Howard Hughes Medical Institute, California Institute of Technology, Pasadena, California, United States of America; 3 Division of Engineering and Applied Sciences, California Institute of Technology, Pasadena, California, United States of America; 4 Division of Humanities and Social Sciences, California Institute of Technology, Pasadena, California, United States of America; Center for Genomic Regulation, Spain

## Abstract

How animals use sensory information to weigh the risks vs. benefits of behavioral decisions remains poorly understood. Inter-male aggression is triggered when animals perceive both the presence of an appetitive resource, such as food or females, and of competing conspecific males. How such signals are detected and integrated to control the decision to fight is not clear. For instance, it is unclear whether food increases aggression directly, or as a secondary consequence of increased social interactions caused by attraction to food. Here we use the vinegar fly, *Drosophila melanogaster*, to investigate the manner by which food influences aggression. We show that food promotes aggression in flies, and that it does so independently of any effect on frequency of contact between males, increase in locomotor activity or general enhancement of social interactions. Importantly, the level of aggression depends on the absolute amount of food, rather than on its surface area or concentration. When food resources exceed a certain level, aggression is diminished, suggestive of reduced competition. Finally, we show that detection of sugar via Gr5a^+^ gustatory receptor neurons (GRNs) is necessary for food-promoted aggression. These data demonstrate that food exerts a specific effect to promote aggression in male flies, and that this effect is mediated, at least in part, by sweet-sensing GRNs.

## Introduction

Metazoan organisms in nature constantly face behavioral choices. Depending on the actions selected, an animal may gain access to potential resources or risk starvation, predation or agonistic interactions. Aggression is an ideal system in which to study how the nervous system makes value-based decisions, as the decision to fight comes with apparent costs and benefits, and requires the assessment of a potential conflict: the detection of attractive resources and competitors who limit access to such resources.

As in many other species, *Drosophila* males exhibit a gender-specific repertoire of stereotyped aggressive behaviors [1]–[9]. Recent studies have identified some of the male-specific sensory signals and their physiological receivers relevant for aggression [1], [2], [4], [10]–[20]. In particular, cuticular hydrocarbon pheromones, such as 11-*cis*-vaccenyl acetate (cVA) [1], [3], [9], [21]–[27] and (*z*)-7-tricosene (7-T) [1], [4], [24], [28]–[32] promote aggression through olfactory [1], [2], [4], [5], [10]–[20] and gustatory receptor neurons [10], [12]–[18], [21]. However, the detection of cues from conspecific males is a necessary but not sufficient condition for aggression: male flies will not fight unless a resource, such as food or females, is present [1], [3], [4], [9], [22]–[24], [26]–[32].

Despite much progress, fundamental questions remain unanswered about how resources promote aggression. In particular, it is widely assumed that flies fight in the presence of food due to competition over a limiting resource or to claim territory for potential reproductive advantages [3], [22], [33]–[35]. However, other explanations have not been excluded. For example, increased aggression in the presence of food could simply be due to an increase in encounter frequency and/or duration between males attracted to the resource, or to an increase in aggressive drive or arousal. Food may also increase locomotor activity, promoting increased encounters and thereby indirectly enhancing aggression. In addition, most previous reports [3], [10], [22], [24], [27]–[29], [31], [32], [36]–[40] measured male-male aggression in the presence of females, which added a potential confound, as presence of females can increase aggression on its own [23], [41], [42]. Finally, it is not clear whether food promotes aggression in a purely permissive or in an instructive manner.

A resolution of these issues would be facilitated by a quantitative analysis of aggressive behavior on variable food resources. Such analyses have been enabled by the development of machine vision-based automated aggressive behavior recognition software [26], [43]–[46]. Here we report on the results of such an analysis, performed in the context of systematic and quantitative manipulations of food resource parameters and analyses of their effects on male-male social interactions. Our results set constraints, in a principled and rigorous manner, on models for how food promotes aggression. We also identify a key component of food and its chemoreceptor that are required for aggression.

## Results

### The effect of food to promote aggression is not due to an increase in male-male social encounters

**Figure 1:**
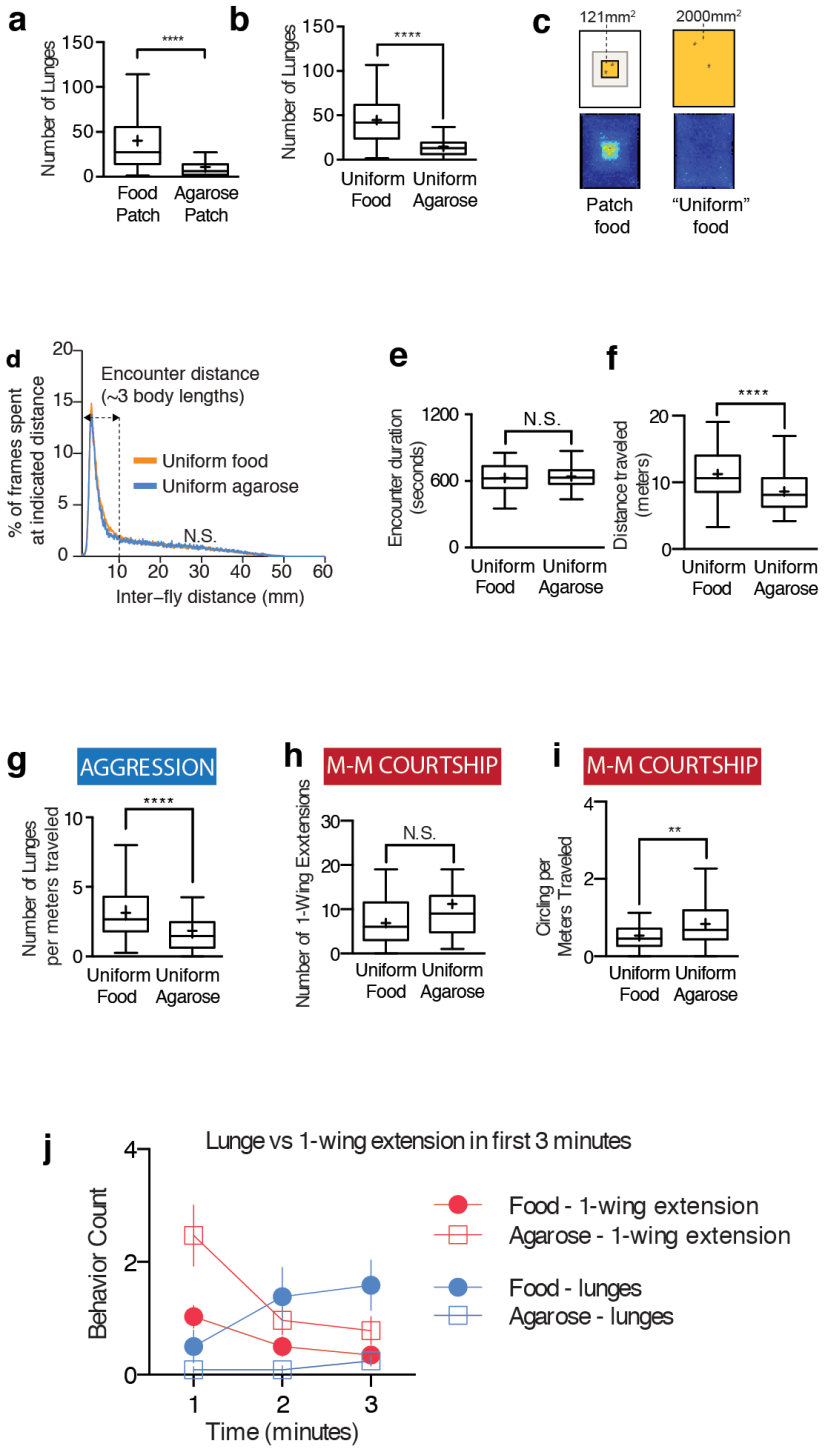
Food is necessary for normal levels of male-male aggression but not male-male courtship and its effects are independent from locomotion or encounter duration. (a) Flies performed more lunges during the observation period in the presence of a 22×22 mm food patch. n = 171, 92 male-male pairs tested for apple juice food patch and agarose patch, respectively. (b) Flies performed more lunges in the presence of arena, which was entirely covered with food. n = 113 and 44, for uniform food and uniform agarose, respectively. (c) Top: Schematic diagram of the aggression assay arenas used. Left side shows the food patch configuration and right side shows the uniform food configuration. A pair of male flies is illustrated at scale for comparison. Bottom: Position heat map shows the average amount of time flies spend in a particular position in the arena. The data shown are averages of multiple pairs of flies (same sample numbers as Figures 1a and 1b). It uses a red-blue color map from MATLAB where deep red is high frequency (60 frames, which is roughly 2 seconds, are the deepest-red) and blue is 0. Every subsequent position heat map is presented in the same manner. On the left, flies are attracted to the patch of food while on the right the uniform food does not lead to attraction to a specific spot in the arena. (d) Uniform food does not change the amount of time flies spend at various distances from each other. The inter-fly distance histogram shows amount of time flies spend (y-axis) at a given distance from each other (x-axis). The distribution is not affected by the presence of food (1-way ANOVA). There is a very prominent peak around 3–4 mm, which ranges from 2 mm (less than 1 body length of flies) to 10 mm (3–4 body lengths), and accounts for around 50% of the 20 minutes assay. The area under the curve from 0 to 10 mm is hereafter referred to as “encounter duration”. The trace is the median trace from 72 and 44 male-male pairs for food and agarose, respectively. (e) Uniform food does not increase encounter duration. Assay is 20 minutes long (1200 seconds). Same number of samples as Figure 1d. (f) Locomotion (distance traveled) in a pair of flies is increased in the presence of food. Same number of samples as Figure 1d. (g) Normalization of aggression by locomotion by dividing the number of lunges by travel distance shows that food significantly increases aggression. Same number of samples as Figure 1d. (h) Number of one-wing extensions is not changed by the presence of uniform food. Manually scored data consisting of n = 17 and 18 pairs for food and agarose conditions, respectively. (i) Normalization of courtship (number of circling bouts) by locomotion shows that food decreases male-male courtship. Same number of samples as Figure 1d. (j) In the first three minutes, food progressively increases aggression (blue circle). In contrast, one-wing extension decreases (red circle). In the absence of food, lunges do not increase or decrease (blue box); courtship decreases (red box). See Table S1 for statistics. Manually scored data of lunges and 1-wing extensions. n = 33 and 33 for food and agarose conditions for lunges. n = 34 and 31 for food and agarose conditions for one-wing extensions.

Previous reports [3], [22], [27], [35], [47] on food's influence on fly aggression used assays with females, leaving open the possibility that food only exerts influence on aggression in the presence of females. Recently, a paper in our laboratory [9], [48] showed that in a small arena without females, food increases aggression in a pair of males. We investigated whether the presence of a central food patch in a bigger arena (as described in [26], [49]) could increase aggression compared to agarose and observed an increase in the number of lunges in the presence of food (Figure 1a, apple juice mixed with 100 mM sucrose and 1% agarose is hereafter referred to as “food”; different from fly culture medium).

Fly aggression assays are typically performed in the presence of a small central food patch [26], [50] or an elevated cup containing food [3], [22], [27], [51], [52], placed in a larger chamber (Figure 1c, left and Figure S4a). Since food is an attractive resource [53], [54], it is possible that food increases aggression by simply increasing the proximity between the two flies due to their attraction to food. This increase in proximity could in turn increase the frequency or duration of encounters between flies. As aggressive interactions between males depend on non-volatile cuticular hydrocarbon pheromones that are detected by contact chemoreceptors [1], [3], [4], [10], [12], [14], [18], [22], [27], [55]–[57], an increase in encounters might enhance aggression indirectly, by promoting pheromone detection. In order to distinguish whether the effect of food to enhance aggression was due to an increased fly proximity on the food patch, we repeated the assays in a modified arena in which the entire surface was covered with a food substrate (Figure 1c and Figure S4b). Control arenas were covered with a uniform layer of agarose. Under these conditions, there was still a clear and significant effect of food to increase the number of lunges (Figure 1b).

To gain further insight into how food affects the proximity of flies and how this may affect the level of aggression, we examined a heat map of fly distribution in the presence of a patch of food and uniform food (Figure 1c). As expected, a central food patch in aggression assays increased the density of flies in this local area (Figure 1c left and Figure S1a), but in an arena containing uniform food, flies were not localized in any particular spot (Figure 1c right and Figure S1b).

To quantify the effects of aggregation on proximity between two flies, we measured the amount of time flies spent at various distances from each other (Figure 1d). This histogram revealed a prominent peak at an inter-fly distance of 3–5 mm, suggesting that flies have a preference to remain within 1–2 body lengths (depending on orientation, average male fly body length is ∼2.5 mm). The height of peak was the same whether uniform food was present or absent (Figure 1d). In contrast, in the presence of a small food patch, there was a small but statistically significant increase in the height of the interaction peak (Figure S1e). This peak likely reflects a preferred interaction distance, as transformation of one fly's position with respect to time by reversing the order (first frame becomes the last frame of the assay) or shifting the order (first frame becomes the 1000th frame) while keeping the other fly's position constant led to a completely different inter-fly distance distribution (Figure S1f and Figure S1g). In order to convert this distribution to a single metric, we integrated the area under the peak between 0 to 10 mm (3–4 body lengths depending on the orientation of the two flies), which we operationally define as “encounter duration”, which accounts for roughly 50% of the time flies spend during the assay. This parameter was not significantly different between uniform food vs. agarose (Figure 1e), further confirming that food is able to increase aggression without affecting proximity and encounter parameters. Encounter duration was a more robust measure of proximity than other measurements of proximity, such as encounter frequency, because encounter duration displayed less variance, was uncorrelated with aggression (Figure S2a) and contained temporal information (i.e. long encounter vs. a short encounter). Taken together, these data indicate that the presence of food can increase aggression independently of any effect to increase the average time that flies spend in proximity to each other.

### Food increases male-male aggression independently of arousal

The foregoing analysis left open the possibility that food might promote aggression by increasing general arousal. One measure of general arousal is locomotor activity [58], [59]. Indeed, a pair of male flies exhibited a small but significant increase in distance traveled in the presence vs. the absence of food (Figure 1f). Because aggression itself involves increased locomotion (Figure S3a) [26], [45], it is not clear whether increased locomotion is a cause or a consequence of increased aggression. Previous studies have addressed this by normalizing the number of lunges to total distance traveled [26], [45]. Normalized for locomotion, food still robustly increased aggression (Figure 1g).

If food increases aggression by increasing general or social arousal, it might also be expected to increase male-male courtship, another social behavior observed in these assays [1], [2], [4], [14], [16], [19], [30], [45], [60], [61]. Male-male courtship is known to be inhibited by male-specific pheromones [1], [19], [62] but it is still observed among pairs of wild-type male flies albeit at low frequency [45], [61], [63]. Unlike male-male aggression, food did not increase male-male courtship, measured by unilateral wing-extensions (Figure 1h) and circling behavior after normalization for distance traveled (Figure 1i).

Male-male courtship occurs predominantly in the first few minutes of a social encounter, and therefore averaging over the entire 20-minute assay might have missed a transient food-dependent increase (Figure S3b). As expected, food increased aggression in the first three minutes (Figure S3c). In contrast, food actually decreased the frequency of one-wing extensions over the first three minutes of the assay (Figure 1j and Figure S3d). Thus, in pairwise male-male social encounters, food selectively enhances aggression but not male-male courtship. These results support the notion that food can specifically increase aggression in a manner that does not reflect a general increase in social interactions.

### The level of aggression depends on the absolute amount of food

**Figure 2:**
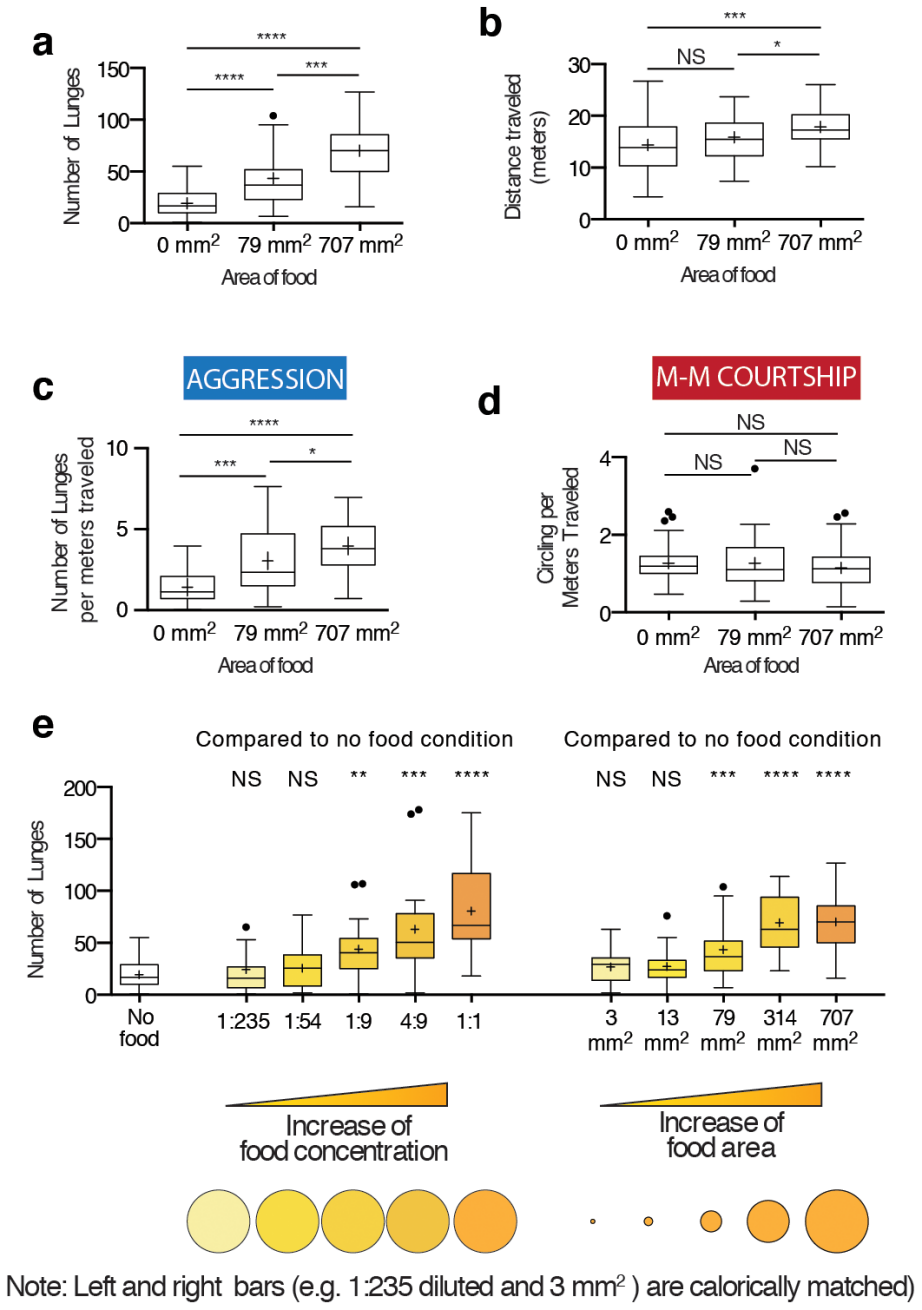
Flies measure the level of total nutrients to increase the level of aggression, rather than the area of food. (a) Aggression increases as the size of food patch increases. See Figure S4 for schematic diagrams of the arena used. n = 41, 39, and 52 male-male pairs for 0, 79, 707 mm^2^, respectively. Same pairs are further analyzed for Figures 2b-d. (b) Locomotion also increases in some cases (0 vs. 707 mm^2^) as the size of food increases. (c) Aggression normalized by locomotion is significantly increased in the presence of food. (d) Male-male courtship normalized by locomotion is not changed by the presence of food. (e) Left: Increasing the concentration of food while keeping the size of food constant (707 mm^2^) increases aggression. Right: Increasing the size of food while keeping the concentration constant also increases aggression. The concentration-dependent increase in aggression is quantitatively similar to the size-dependent increase in aggression. The absolute nutritional content remains the same between the left and the right (1∶235 = 3 mm^2^, 1∶54 = 13 mm^2^, etc). Some of the data in E are the same as those used in A and are replotted here for comparison purposes. n = 41, 22, 16, 29, 28, 31, 36, 37, 39, 27, and 52 male-male pairs from left to right.

If food specifically enhances aggression, how do flies measure it? The answer to this question sets constraints on the sensory systems that are involved, and ultimately how the brain uses this information to guide the decision to fight. We first examined the effect of changing the area over which food (at a fixed concentration) is distributed, using a modifiable arena (Figure S4c). Consistent with previous reports [22], [27], we observed a dose-dependent relationship between the size of the food patch and the level of aggression (Figure 2a). Next we investigated whether this dose-dependent increase was due to an effect on either proximity, arousal, or general social interactions. Although, we observed a slight increase in locomotion as the size of the food patch increased (Figure 2b), this enhanced aggression was seen even when normalized by locomotion (Figure 2c). Furthermore, the inter-fly distance distribution was not changed by any of the differently sized food arenas that were tested (Figure S5b). Unlike aggression, male-male courtship showed no change in response to the change in the amount of food (Figure 2d), suggesting that the dose-dependent effect of food does not reflect a general increase in social interactions.

Previous studies did not distinguish whether the increase in aggression caused by increasing the size of food patch was due to an increase in area, total food amount or both [22], [27]. We therefore investigated whether changing the concentration of food while keeping the arena area constant would yield a similar result. Indeed aggression in a fixed-size arena increased as the concentration of food increased (Figure 2e left). In fact, when we compared the level of aggression in the cases where the areas of food were different (Figure 2e right) but the caloric content was matched, the level of aggression was indistinguishable (see Figure S5a for side-by-side comparisons). These data are incompatible with the notion that flies assess the quality of food in the context of aggression by using a physical dimension of food territory, such as area or perimeter circumference. Instead, these results suggest that the level of aggression depends upon the absolute amount of food in the substrate.

### Flies decrease fighting when food exceeds a certain threshold

**Figure 3:**
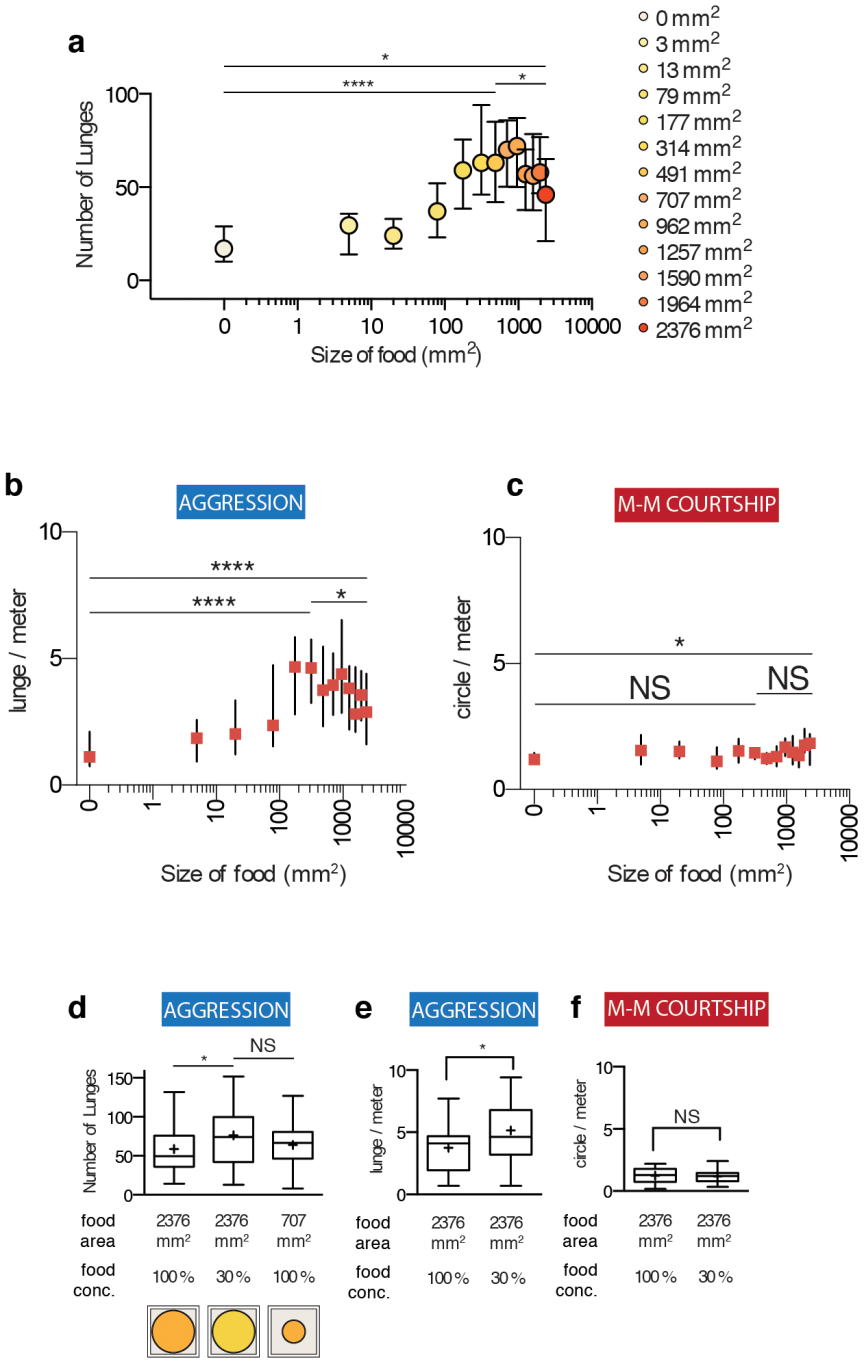
Flies decrease the level of aggression as the availability of food resource increases. (a) The relationship between aggression (y-axis) and the amount of food (x-axis). Aggression initially increases from 0 mm^2^ to 707 mm^2^ and decreases as the size of food increases further. In particular, aggression observed with the largest size tested 2376 mm^2^ is significantly lower than 707 mm^2^ after correcting for multiple comparisons. Some of the data are the same as those used Figure 2 and are replotted here for comparison purposes. n>28 male-male pairs for each condition tested. Pairs are further analyzed for Figures 3b and 3c. (b) Aggression normalized by locomotion shows the same initial increase and subsequent decrease (See Table S3 for pair-wise comparison statistics). (c) Male-male courtship normalized by locomotion shows no increase or decrease (See Table S4 for statistics). (d) The decrease in aggression seen in the largest food patch tested (left, 2376 mm^2^) can be reversed by decreasing the concentration of food to 30% (middle). Calorically, this condition is equivalent to 707 mm^2^ food patch with 100% concentration of food (right) and the amount of aggression is indistinguishable. The 707 mm^2^ food patch data replotted for comparison purposes. n = 32, 31, 86 male-male pairs from left to right. (e) The increase in aggression by dilution of food is significant after normalization for locomotion. n = 32, 31. (f) There is no change in courtship caused by the dilution of food. n = 32, 31.

The foregoing experiments show that aggression requires a minimal amount of food, and scales as the quantity of food increases. If aggression is driven by competition over food, then aggression should decrease at some point, if the food becomes available in excess, as it is seen in many other species [64], [65]. Indeed, previous studies showed that a very large area of food can decrease aggression in comparison to an intermediate area of food [22]. We confirmed these findings in our setup by testing 5 additional larger food patches with areas >707 mm^2^. Under these conditions, we observed a gradual decrease in aggression as the area of the food patch was increased to 2376 mm^2^, the largest size tested (Figure 3a and Figure SS4c).

It was previously suggested that the decrease in aggression observed may be due to the increased energetic cost of defending a greater territory or a larger food patch [22]. However, given our finding that fly aggression depends on the absolute amount of food rather than the area of food, it remained a possibility that the decrease in aggression was also caused by a greater quantity of food. Indeed, when we decreased the concentration of food in the largest arena (2376 mm^2^ arena) from 100% to 30%, aggression was increased to a level equivalent to that in a smaller (707 mm^2^) but nutritionally identical arena containing 100% food (Figure 3d). This increase in aggression was still significant after normalization for locomotion (Figure 3e and Figure S5c), while male-male courtship did not show any increase (Figure 3f). These data further support the idea that flies tune their level of aggression as a function of the absolute amount of food available. Aggression is enhanced as the amount of food is increased to a certain point, and decreases as the amount of food is increased above that amount.

The dose-response relationship we observed above suggested that there could be a continuous relationship between the amount of food and aggression. This would imply that the role of food may be instructive rather than purely permissive. Nevertheless, using the Kruskal-Wallis test, we were only able to resolve a few statistically distinct groups among the different sizes of food tested, due to the high pair-to-pair variability in the amount of fighting (Table S3). One shortcoming of using Kruskal-Wallis test is that since it treats groups being tested as categorically distinct, as the number of groups increases, Bonferroni corrections for multiple comparisons reduce statistical power to resolve small differences. For instance, among the 13 different sizes of food we tested, there were 78 comparisons made, and after correcting for multiple comparisons, only a few points were statistically significantly different from each other, despite the fact that when individually tested in a pair-wise manner, many more were significantly different (See Table S3).

As an alternative approach to this problem, since the amount of food is a continuous rather than a discrete variable, we performed a curve-fitting analysis to model the relationship between food quantity and aggression. The simplest possible model to test whether the data we observe has an increasing phase and a decreasing phase is the quadratic function (Figure S6a). We ran an ordinary least squares estimation method, a form of regression analysis, among quadratic functions, to find the coefficients *β_0_, β_1_*, and *β_2_*, which best fit the data. The results (Figure S6b) suggested that 1) There is a non-random relationship between the amount of food and aggression and 2) there is an inverse-U shaped relationship between the amount of food and aggression. That is, since the coefficient *β_0_* is significantly different from 0, it implies that the as food increases, aggression goes up until it reaches a certain threshold and then goes down. The 99% confidence intervals for the coefficients *β_0_, β_1_*, and *β_2_*, show that the model predicts an *X*-intercept of 14 to 26 (14 to 26 lunges when there is no food) and an inverse-U shape (99% confidence interval for *β_0_* is bound within negative values). The results of the analysis were statistically significant for the joint F-test for coefficients *β_0_, β_1_*, and *β_2_*, which suggests that there is a non-random relationship between aggression and the amount of food. Since the coefficient *β_0_* is significantly different from 0, a quadratic function yielded a higher fit to the data than a linear function (Figure S6c). This analysis suggests that aggression exhibits a continuous increase and then a decrease as the quantity of food is increased rather than having an all-or-none effect.

While aggression showed an inverse U-shaped curve in response to increasing amount of food (Figure 3a, 3b, Figure S6b and Table S3), locomotion (Figure S5c and Table S2) and male-male courtship (Figure S5d and Table S4) showed no such patterns, suggesting that the biphasic response is specific to aggression. Encounter duration was slightly different when compared to the no-food conditions (Figure S5d) although the overall inter-fly distance distribution remained unchanged (Figure S5b). These data confirm and extend the results of the previous finding [22], but are inconsistent with their interpretation that a larger size of food decreases aggression due to the increased energetic cost of defending a larger territory. Instead, we favor the idea that aggression between flies reflects competition over limiting amounts of food resources, which can be partially overcome when nutrients exceeds a certain threshold.

### Flies display territorial behavior

Territorial behavior refers to overt or implied defense of an area by one or a group of animals at the exclusion of others [66]. Although the term territoriality is frequently used when referring to aggression in *Drosophila* [3], [22], previous studies have not distinguished between the defense of a territory (territoriality) from the defense of a resource per se [6], [35]. To investigate this issue, we observed in more detail the spatial distribution of a pair of flies with respect to food resources of different areas.

**Figure 4:**
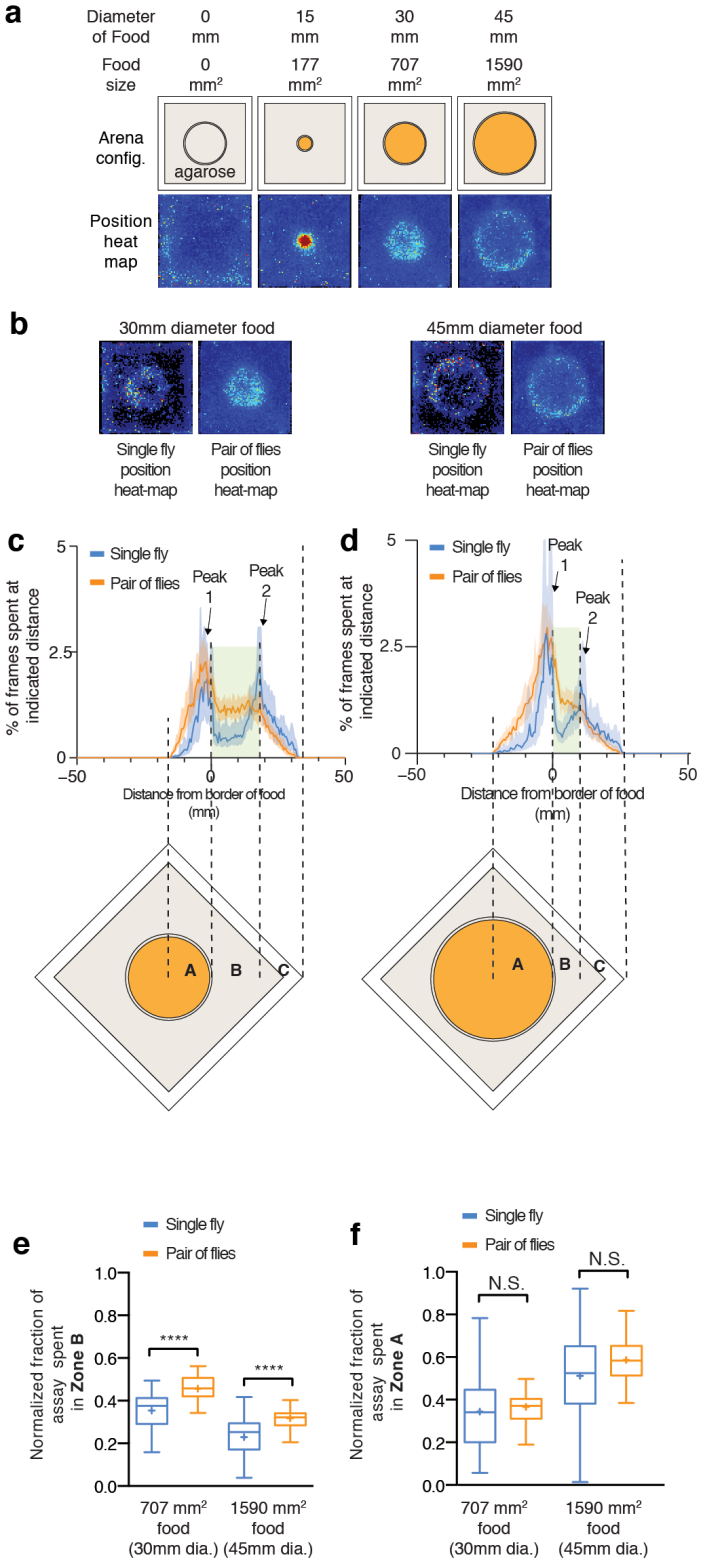
Flies display territorial behavior. (a) Top row: Schematic diagrams show the arenas with different size of food being used. Bottom row: Position heat-map of a pair of flies presented with different sizes of food. The heat-maps display two features: 1) flies spend a lot of time on top of food and 2) they spend a lot of time near the border of the food area. n = 41, 29, 86 and 41 male-male pairs from left to right. (b) Position heat map compares the distribution of flies on 30 mm and 45 mm diameter food when there is only 1 fly in the arena (left) and when there are two flies (right). 2-fly data from one experiment are individually averaged. n = 30 and 52 for 30 mm diameter food, single and pairs of flies, respectively. n = 25 and 41 for 45 mm diameter food, single and pairs of flies, respectively. The pairs are further analyzed in Figures 4c – 4f. (c and d) These histograms show the amount of time flies spend at different distances from the border of 30 mm (c) food and 45 mm (d) patch. The schematic diagrams of the behavioral setups are overlaid for visualization. Briefly, the x-axis is aligned so that 0 denotes the border of food patch while negative values indicate the distance inward from food border (inside the food patch) and positive values indicate the distance outward from the food border (outside of food patch). The blue line denotes when there is a single fly in the arena while the orange line denotes when there is a pair of flies. Lines indicate the median while shaded area denotes the interquartile range. (e) Presence of another fly increases the amount of time flies spend in Zone B (“interaction zone”) for both 30 mm and 45 mm food patches. (f) Presence of another fly does not change the amount of time flies spend on the food patch (Zone A).

As mentioned earlier, flies preferentially occupy the area where food is present (Figure 1b and 4a). In addition, we observed that as the area of the food patch was increased, the position heat map showed an apparent circular “donut” shape (Figure 4a), suggesting an increased preference of flies to remain near the periphery of the food patch. This observation suggested that flies may defend the perimeter of the food, rather than the entire food resource, when the size of the patch is large.

To distinguish whether this phenomenon was related to aggression, or simply reflected an innate preference of flies to occupy the boundary of a food patch, we compared the distribution of single flies and fly pairs for two different sizes of food patches (Figure 4b). In order to quantify these distributions with respect to the food patch area, we measured the amount of time flies spent as a function of the distance from the food patch border patches, and aligned the histograms to the border defined as 0 mm (Figures 4c and 4d). In both 30 mm and 45 mm diameter patches, we observed two peaks defining three zones in the histograms, which we refer to as Zones A, B, and C (Figures 4c and d, lower). Zone A comprised the food patch itself and exhibited a peak in the fly distribution at the border. Zone B comprised the area between the food border peak and a second peak, located approximately 15–20 mm from the outside edge of the arena. Zone C comprised the perimeter area of the arena. Since Zone A was the area occupied by the food patch, fly occupation of this area simply reflected their natural attraction to food. Zone C could, in part, reflect thigmotactic tendencies of flies [67], [68], since in the absence of food, a similar peak around 15–20 mm from the edge of the arena was also observed (Figure S7a). To investigate whether these experimental peaks were different from a random distribution, which would be expected if flies behaved as if they were randomly moving particles, we calculated a random distribution from the area in the bins at each indicated distance from the food border and compared it to the experimental distribution (Figure S7b). These comparisons revealed that in the absence of a food patch (blue line), flies behaved similarly to randomly moving particles (teal colored line). In contrast, in the presence of a 30 mm diameter food patch, fly positions (orange) were not randomly distributed.

In both single and paired fly experiments, there were two peaks dividing these three zones in both 30 mm diameter (Figure 4c, blue for single fly and orange for paired fly experiments) and 45 mm diameter food patches (Figure 4d). Nevertheless, we observed a noticeable difference in the distribution of flies within Zone B. Pairs of flies appeared to spend more time in this zone than did single flies. To quantify these differences, we calculated the area under the curves in Zone A and Zone B for single vs. paired flies. Single male flies spent significantly less time than did flies in pairs in Zone B for both 707 mm^2^ and 1590 mm^2^ food patches (Figure 4e). In contrast, when we calculated the amount of time flies spent in the food area (Zone A), we found that the presence of an opponent male made no difference (Figure 4f). These data indicate that the presence of an opponent does not enhance attraction to food; instead it only increases the amount of time flies spend in the area just outside the food border, suggesting that fighting flies adopt a “perimeter defense” strategy. These data are consistent with the notion that when the size of the food patch is large (Figure 4a, 177 mm^2^ vs. 1590 mm^2^), *Drosophila* males fight over access to a food-containing territory, rather than just over the food resource itself.

### Sucrose is sufficient to promote aggression

**Figure 5:**
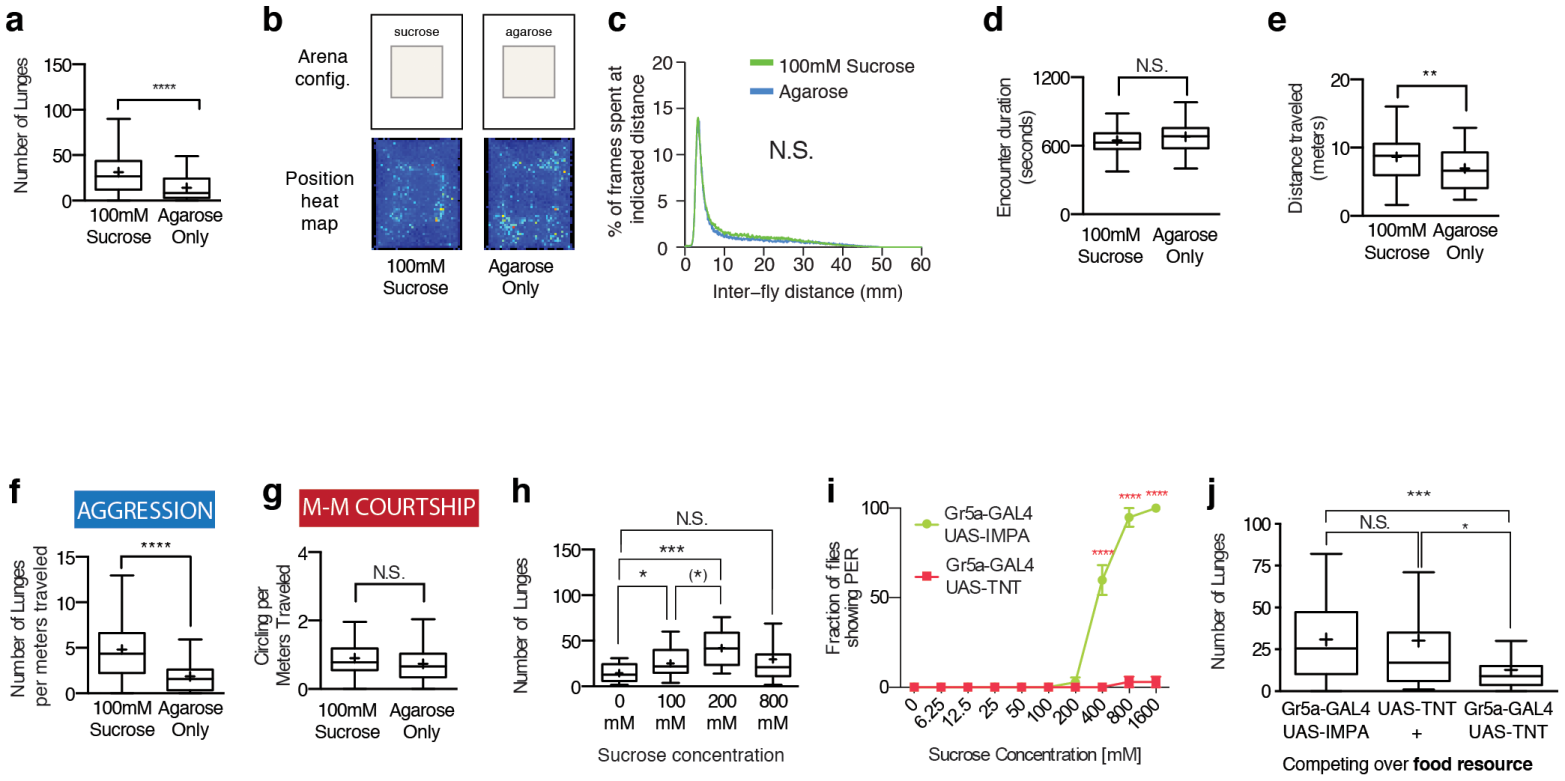
Flies use sweet-sensing Gr5a^+^ GRNs to detect the concentration of sucrose in the food and tune the level of aggression accordingly. (a) 100 mM sucrose is sufficient to increases aggression. (b) Sucrose does not cause attraction, as it does not lead to an apparent change in the position heat map. n = 100 and 60 for 100 mM sucrose and agarose, respectively. Pairs are further analyzed from Figures 5b-5g. (c) Presence of sucrose does not change the amount of time flies spend near each other. (d) Encounter duration does not change in the presence of sucrose. (e) Sucrose increases locomotion. (f) Sucrose increases the number of lunges per meters traveled, which implies that the increase in aggression is not merely due to increased locomotion. (g) Sucrose does not change the number of circling per meters traveled. (h) Changing sucrose concentration increases and decreases aggression. The level of aggression is increased from 0 to 200 mM but becomes indistinguishable from no food condition at 800 mM. (*): 100 to 200 mM difference is significant when individually compared (P<0.05) but not after corrections for multiple comparisons. n = 32, 23, 10 and 26 from left to right. (i) Inhibiting the sugar-sensing Gr5a^+^ GRNs by expressing TNT decreases sucrose sensitivity (n = 3 and 3 for both genotypes. Each replicate has 10 male flies to calculate fraction of responders). (j) Inhibiting the sugar-sensing Gr5a^+^ GRNs by expressing TNT decreases food-promoted aggression compared to genetic controls. n = 36, 41, and 32 from left to right.

Foregoing data suggested that flies may use their chemosensory systems to measure the absolute nutritional content of the food to tune the level of aggression. Apple juice and fly culture food are complex mixtures containing a variety of odorants and tastants [69]–[71]. One obvious indicator of nutritional content in natural food resources is the concentration of sugar. Therefore, we tested whether pure sucrose, present in fly culture medium and food mix used in our experiments, would be sufficient to increase aggression in the absence of any other food component. Surprisingly we found that a small patch of 100 mM sucrose (see Figure S4e), comparable to concentrations found in fruits [33] and in laboratory fly food medium [70], was sufficient to promote aggression to a level comparable to that observed using the food substrate (Figure 5a and Figure S8d). Similar to uniform food, the ability of sucrose to increase aggression was not due to a difference in the encounter duration, because the presence of a patch of sucrose neither changed the overall distribution of the flies (Figure 5b), nor changed the encounter duration (Figure 5c and 5d). The presence of sucrose increased locomotion (Figure 5e), but the increase in aggression caused by sucrose remained significant following normalization to distance traveled (Figure 5f). In contrast, male-male courtship was not increased (Figure 5g). Thus pure sucrose can mimic the effect of food to increase aggression.

To examine the dose-dependency of aggression on sucrose, we compared the number of lunges in 100, 200 and 800 mM sucrose (Figure 5h, see Figure S4e). Similar to the results obtained with food (Figure S8d), we first saw an increase in aggression when we increased the concentration of sucrose from 100 to 200 mM. Moreover when we further increased the level of sucrose to 800 mM, the level of aggression was no different from the control condition (Figure 5h). Taken together, these data suggest that sucrose exhibits a bi-modal influence on aggression that is qualitatively similar to that seen with food.

### The activity of sugar sensing Gr5a^+^ gustatory receptor neurons is required for aggression

Previous work has shown that several subpopulations of fly gustatory receptor neurons play a role in male-male aggression and male-male courtship via detection of pheromones [1], [4], [10]–[18]. Sucrose is known to be detected by Gr5a^+^ GRNs in the fly gustatory system [21], [24], [25]. However these GRNs have previously only been implicated in the context of feeding and proboscis extension behaviors [24], [28], [29], [31], [32]. Because we found that sucrose is sufficient to promote male-male aggression, we investigated whether the activity of Gr5a^+^ GRNs is required for male-male aggression on food. To test this, we silenced the neurons by expressing tetanus toxin light chain (TNT) [34] under the control of the Gr5a-GAL4 promoter [31].

First we verified that silencing the Gr5a^+^ GRNs via expression of TNT reduced sucrose sensitivity by performing proboscis extension reflex (PER) assay (Figure 5i), as described previously [24], [28], [29], [31], [32]. Next we tested the effect of silencing Gr5a^+^ GRNs on aggression and found that the activity of Gr5a^+^ GRNs is necessary for aggression on food (Figure 5j). Importantly, flies whose Gr5a^+^ GRNs were silenced could still perform aggression at a level comparable to the genetic controls in the presence of females, suggesting that the effect of silencing Gr5a^+^ GRNs did not merely impair the ability to fight (Figure S8a). We confirmed that we could get the same result of reduced aggression in the presence of food using another effector, UAS-Hid [42], which was shown to disrupt the function of Gr5a+ GRNs [44], [46] (Figure S8b). Since food contains various gustatory and olfactory cues [33] that are not detected by Gr5a^+^ GRNs, these data suggest that detection of sweet tastants plays a permissive role in food-induced aggression.

Finally, we investigated whether increasing the activity of Gr5a^+^ GRNs would suffice to increase aggression. To do this, we expressed different effectors, that increase the neuronal activity in Gr5a^+^ GRNs, including UAS-DTRPA1, UAS-TRPV1, UAS-NaChBac tub-Gal80ts, UAS-ChR2 and UAS-ReACh [10], [28], [36]–[40]. However, none of the effectors increased aggression in the absence of food (Figure S8c; UAS-dTrpA1, UAS-ChR2 and UAS-ReACh data not shown). This was true even for TRPV1, a cation channel activated by the ligand capsaicin, which was added to the agarose substrate in order to ensure activation of Gr5a GRNs on the tarsae (Figure S8c). These data suggest that although Gr5a^+^ GRNs are necessary for normal levels of food-induced aggression, they are not sufficient to increase aggression in the absence of food.

## Discussion

In nature, when confronted with another animal, a male has to decide whether to engage in social behavior and if so, whether to engage in aggression or courtship. Understanding how information processing in the brain controls such behavioral decisions is a fundamental problem in neurobiology. An essential first step in this framework is to identify the relevant sensory cues to a particular behavior and neural circuits, which process these inputs.

Intraspecific aggression is an innate social behavior observed in many species. The presence of either food or a mating resource is fundamental to releasing aggression, as a link between these resources and aggression has been observed in many species, such as primates [41], mice [43], birds [47], fish [48], squid [49], spiders [50], ants [51], cockroaches [53], and flies [3], [22], [27], [55].

In flies, correlations have been observed between an increased probability of aggressive encounters and the presence of females or various food substrates [1]–[4], [16], [19], [20], [22], [23], [27]. Nevertheless, as most studies investigated aggression in the presence of both food and a female, until recently [1], [9], no study has compared the level of aggression with food vs. no food in the absence of females. Furthermore, no study has distinguished whether attractive resources directly promote male-male aggression in *Drosophila*, or rather promote this behavior indirectly simply by increasing the proximity and therefore the probability of encounter between competing males [1], [3], [4], [6], [22], [30], [72]. In addition, it was not clear whether food increased all social behaviors or specifically increased aggression. Resolving these issues is fundamental to studies of aggression in all animals.

Here we confirm that in flies, food can increase aggression relative to an agarose substrate, in the absence of females [2], [5], [9]. Furthermore, we provide evidence that this effect is not due to an increase in the proximity of flies to each other: food covering the entire surface of the arena does not increase encounter duration but nevertheless increases aggression. Our data also indicate that although food slightly increases locomotor activity, its effect to increase aggression is still significant even after normalizing for locomotion. In contrast to aggression, male-male courtship is unchanged or even somewhat decreased, by the presence of food. Taken together, these data suggest that food specifically promotes aggression.

Previous studies have demonstrated that there is a food patch size-dependent increase in aggression in flies [22], [27]. But it was unclear from these studies whether the flies were responding to an increase in the amount of food, or rather the area of food. We systematically compared increases in both food area at a fixed concentration, and increased food concentration in an arena of fixed area. Both manipulations increased the amount of aggression (up to a certain point), indicating that the relevant factor is the absolute amount of food, rather than the area over which it is distributed. Importantly, above a certain amount of food, aggression is decreased, while decreasing the concentration of food in the same-size arena increased aggression. These data reveal a dose-response relationship between food and aggression, suggestive of competition.

Since we observed multiple incremental steps in the level of aggression as the amount of food was increased, food seems to play an instructive role in promoting aggression rather than a purely permissive role: in the latter case there would be only two statistically distinguishable levels of aggression, high when there is any amount of food and low when there is no food. Nevertheless, because there is a large amount of pair-to-pair variation in aggression, the change in aggression can only be detected between large changes in the amount of food. It is unclear why a male fly, whose length is approximately 2.5 mm, continues to increase aggression until the diameter of the food patch reaches 30 mm, and only decreases aggression slightly when the diameter of food exceeds 50 mm (a circular patch 25× length of the fly body). The exact mechanism by which flies “measure” the absolute amount of food to tune the level of aggression is unclear.

We also found that sucrose, which is present in many fruits, fly medium, and the food in our assay, mimic food's effects on aggression [70]. There is a dose-dependent increase in aggression and eventual decrease after the amount of sucrose exceeds a certain amount, similar to the effect of food. By inhibiting Gr5a^+^ GRNs, the sweet-sensing gustatory receptor neurons in flies, we showed that the sugar-sensing gustatory receptor neurons play a permissive role in aggression promoted by food. Artificial activation of Gr5a^+^ GRNs failed to increase aggression however. This result, taken at face value, would seem to suggest a permissive and not instructive role for sugar in aggression, in seeming contradiction to the result of our dose-response studies. The reasons for our failure to show that artificial activation of Gr5a^+^ GRNs is sufficient to increase aggression may be technical or biological. Technical reasons could include an inability to activate Gr5a^+^ GRNs to a critical threshold necessary for aggression, perhaps due to a depolarization block [39]. Alternatively, Gr5a^+^ GRNs may be required to detect the presence of sugar, but the calculation of relative resource value may require higher order circuits. It is worth noting that sucrose is attractive to egg-laying females [73], much like various types of fruits [22]. This suggests the possibility that male flies may compete over food not only to gain access to nutrients, but also to locations where egg-laying females are present. Consistent with this idea, food also increases male-female courtship [74].

A potential caveat regarding our experiments with Gr5a^+^ GRNs is that our GAL4 driver may also be expressed in pheromone-sensing GRNs. However, the available data do not support that possibility. Previous studies showed that Gr5a-GAL4 do not overlap with markers for pheromone-sensing GRNs (ppk23, ppk25, and fru^M^) [10], [14], and that Gr5a^+^ GRNs did not respond to male pheromones [20]. Furthermore, disruption of Gr5a^+^ function does not decrease aggression when the aggression promoting resource is females instead of food (Figure S8a), nor does it produce any effect on courtship or social behaviors [18], [75]. Finally, disruption of Gr5a^+^ GRNs function decreases aggression in the presence of sucrose (Figure S8c). Taken together, these data strongly argue against the possibility that the requirement for Gr5a GRNs in aggression on food is due to a role in pheromone rather than sugar detection.

Aggression in flies is typically considered to be “territorial” [3], [22]. However there is a difference between the defense of a territory containing a particular resource, and the defense of the resource itself: a bird may defend a nest or defend a larger area in which the nest is located. The available data do not distinguish between the two in the case of *Drosophila*. We observe that although single flies exhibit an innate attraction to food, in the presence of another male, they spend more time just outside the perimeter of the food area. Correspondingly, most fighting occurs in the perimeter surrounding the food area. This “doughnut” effect is most apparent when the food patch becomes larger than 20 mm in diameter; in smaller diameter arenas, fighting occurs throughout the food patch.

These observations are consistent with (but do not prove) the idea that when the area of the food patch exceeds a certain size, flies adopt a “perimeter defense” strategy. Since such a strategy is the most energetically efficient way for a fly to prevent occupancy of a large food patch by its competitor, these results suggest that aggression in flies may indeed involve territorial defense. Nevertheless, we cannot formally exclude the possibility that flies fight at the patch perimeter simply because they prefer to occupy this area.

Taken together, our experiments show that food promotes aggression in flies, in a manner that is not simply an indirect consequence of arousal, aggregation on food, or a general increase in social interactions. Flies increase and decrease the amount of aggression depending on the amount of food available, which is suggestive of competition over a limiting resource: aggression declines when the resource exceeds a certain threshold. The detection of this resource requires gustatory sugar receptor neurons that express *Gr5a*, consistent with the idea that it is the perceived caloric value of the resource that promotes aggression. Finally, flies exhibit a “perimeter defense” strategy, which is suggestive of a function for aggression to prevent the opponent from gaining access to a resource-rich territory. Together, these data offer new insights into the control of aggression in flies by food, which may apply to other species as well.

## Materials and Methods

### Behavioral assays and analysis

Behavioral assays were performed using 3–7 day old male flies that were raised in isolation. Group-housed flies were used in experiments shown in Figure 5, because group-housed male flies show female-induced aggression, unlike single-housed flies, which show a high level of baseline aggression even without females. In all experiments involving the Gr5a-GAL4 flies and their genetic controls, comparisons were made on equivalent genetic backgrounds. Most experiments were performed in a 40 mm×50 mm behavior chamber previously described [26] or the new 70 mm×70 mm chamber (Figure S4c) that allowed us to test different amounts of food. Briefly, two males were introduced into the chamber by gentle aspiration, recorded for 20 min, and behavioral data were extracted from the recorded videos using CADABRA software or directly from MATLAB. Temperature and humidity were kept around 25°C and 40–50% R.H. and all experiments were performed around the activity peak of flies, either from 7 am to 3 pm or 7 pm to 3 am. As flies have to be able to see in order to fight, all experiments were performed using a ring-shaped strip of white LEDs to illuminate the behavioral chambers. From these analyzed movies, we extracted several parameters, such as position of flies with respect to food, frame by frame inter-fly distance, distance traveled, number of lunges performed, and number of circling behaviors performed. These parameters were manually checked to make sure that the tracking algorithm was reporting with high fidelity. For male-male one-wing extensions, behavior was scored manually as we found that CADABRA was unable to report an accurate count of male-male one-wing extensions. Thus we used number of circling bouts instead of number of one-wing extensions to measure male-male courtship, except to show that food does not increase male-male courtship. All of the different chambers used can be seen in schematic drawings in Figure S4.

### Fly stocks and rearing conditions

All fly stocks were reared in plastic vials containing yeast, corn syrup, and agar medium at 25°C, 60% humidity, and a 12-h light∶12-h dark cycle. Newly eclosed males were reared either individually (single housing) or at 10 flies (group housing) per vial [2.4 cm (diameter)×9.4 cm (height)] for 3 or 7 days before performing the behavioral assay. Wild-type Canton-S (CS) flies were used for all experiments unless otherwise indicated. Gr5a-GAL4 flies were a gift from the John Carlson Lab. UAS-TNT and UAS-IMPTNT flies were acquired from Bloomington. UAS-Hid flies were a gift from Joel Levine Lab. UAS-Shi^ts^ flies were flies were a gift from obtained from the Gerald Rubin Lab [76]. All transgenic flies used, such as the Gr5a-GAL4, UAS-TNT, UAS-IMP, UAS-Hid, UAS-nlsGFP UAS-Shi^ts^ were backcrossed for 6 generations into the CS background. All behavioral assays were performed using males carrying the wild-type X chromosome.

### Statistical analyses

Most of the behavioral data were nonparametrically distributed; thus, only nonparametric tests were used to test for statistical significance. Mann-Whitney U tests (for pairwise comparisons) and Kruskal-Wallis analysis of variance (ANOVA; for comparisons among >2 groups) were applied. Significant difference among groups detected by Kruskal-Wallis ANOVA was analyzed using Dunn's *post hoc* test (with corrections for multiple comparisons) to identify groups with statistically significant differences. Two-way ANOVA was applied for comparisons among histograms.

Boxplots: lower and upper whiskers represent 1.5 interquartile range (IQR) of the lower and upper quartiles, respectively; boxes indicate lower quartile, median, and upper quartile and the cross indicates the mean. p values in all Figures represent Kruskal-Wallis one-way ANOVA followed by Mann-Whitney U tests with Bonferroni correction when there are more than two groups for comparison. p values are abbreviated using asterisks. *: p<0.05, **: p<0.01, ***: p<0.001, ****: p<0.0001, N.S. (not significant): p>0.05.

## Supporting Information

Figure S1 **Proximity between two male flies is changed by the presence of a small food patch but not by uniform food.** (a) In the presence of a small food patch, there is clear attraction to the center of the arena. n = 171 and 92 for food patch and agarose patch, respectively. n = 72 and 44 for uniform food and uniform agarose, respectively. The pairs are further analyzed for all of Supplemental Figures 1. (b) In the presence of food, which covers the surface of the arena uniformly, there is no change in the distribution of the flies with respect to the center of the arena. (c) Quantification of the data in (a) and (b): Median distances from the center of the arena are changed in the presence of a small food patch. (d) Inter-fly distance histogram shows that the presence of a small food patch slightly changes the distribution compared to the absence of food. (e) Sum of the encounter (inter-fly distance <10 mm) duration shows that the presence of a small patch of food slightly increases the amount of time flies spend within 10 mm of each other. (f) Left: Same data as (d) replotted for comparison. Middle: Shows the same data as Left after transformation of the position of one fly with respect to time by flipping the order (first frame becomes last frame and vice versa). Transformation shows that flies are naturally attracted to the center of the arena but the prominent encounter peak is not present, suggesting that the peak depends on the coordinated positioning of two flies. Right: Shows the results of similar transformation as Middle but instead of flipping the order, 1000 frames were added to shift one fly's position with respect to time. (g) Left: Same data as Figure 1D replotted for comparison. Middle and Right: Transformation as performed in (f) shows that the presence of uniform food does not change the position of flies and that the prominent peak in inter-fly distance histogram is likely due to the natural interaction distance of flies.
(TIF)

Figure S2 **Encounter duration is an independent measure of aggression.** (a) Encounter duration, the amount of time flies spend within 10 mm of each other, shows no correlation (*r* = 0.018) with the number of lunges. Most of the points lie near the 600 seconds (50% of the assay) regardless of the number of lunges observed. n = 204 x, y pairs. (b) Encounter frequency, the number of times flies come within 10 mm of each other, shows a weak correlation (*r* = 0.365) with the number of lunges.
(TIF)

Figure S3 **Food promotes aggression and not courtship.** (a) Left: Aggression (number of lunges, y-axis) is linearly correlated with locomotion (*r* = 0.69, travel distance in meters on x-axis). Right: Courtship (number of circling) is linearly correlated with locomotion (*r* = 0.45). n = 171 male-male pairs. (b). Behavioral choice between male-male courtship and male-male aggression develops in the first three minutes of the assay and remains stable. Left: Aggression increases slightly over time in the presence of food (orange). No change is observed in the absence of food (blue). n = 113 for uniform food and 44 for uniform agarose. Right: Male-male courtship (one-wing extension) decreases slightly over time in the presence of food (orange) and without food (blue). One-wing extension data were manually scored. n = 18 and 17 for uniform food and agarose, respectively. (c) Presence of food increases aggression in the first three minutes of the assay. Manually scored lunges for male-male pairs, n = 33 and 33 for food and agarose conditions. (d) Presence of food decreases male-male courtship (one-wing extensions) in the first three minutes of the assay. Manually scored one-wing extensions for male-male pairs, n = 34 and 31 for food and agarose conditions for one-wing extensions. (e) Presence of food decreases locomotion in the first three minutes of the assay. n = 34 and 31 for food and agarose.
(TIF)

Figure S4 **Schematic diagrams of all of the arenas used in behavioral assays.** (a) Patch arena: 11 mm×11 mm food patch is used and compared with agarose. Surrounding the food patch there is an area with agarose. The arena is 40 mm×50 mm. (b) The uniform arena has the entire surface covered with either food or agarose. (c) An arena with concentric rings allows for testing of multiple sizes of food with diameters. The food patch is surrounded by agarose, which is surrounded by a small plastic base. The entire arena is 70 mm×70 mm. (d) Experiments with the sucrose patch were performed with either sucrose or agarose in a 22 mm×22 mm square area in the middle of the arena. (e) Experiments testing different sucrose concentrations (0, 100, 200, 800 mM) were performed with 707 mm^2^ patch of sucrose. (f) Experiments testing female-induced aggression were performed with 40 mm×50 mm arena with a dead female on top of an agarose patch in the middle.
(TIF)

Figure S5 (a) The absolute amount of food, rather than concentration or area of food, determines the level of aggression (1∶235 dilution of food with 707 mm^2^ area is equivalent to a 3 mm^2^ food patch, etc). Every dilution–size pair is statistically indistinguishable from the other condition. The data are replotted from Figure 2e for comparisons. (b) Inter-fly distribution shows the pattern of inter-fly distance does not change over 13 different sizes of food patch ranging from 0 to 2376 mm^2^ does not change the pattern of inter-fly distance (1-way ANOVA). n>28 for all conditions. (c) Locomotion shows little to no change as the size of food changes from 0 to 2376 mm^2^. See Table S2 for details. n>28 for all conditions. (d) Encounter duration shows no change as the size of food changes from 0 to 2376 mm^2^. See Table S5 for statistics. n>28 for all conditions.
(TIF)

Figure S6 **Aggression shows biphasic response to the amount of food.** (a) Functional form being tested for curve-fitting analysis. (b) Curve-fitting the quadratic function of the form in (a) shows that there is an increasing and decreasing pattern. Left: Scatter plot of the experimental data (n = 493). x-axis is diameter of food and y-axis is number of lunges. Right: Each dot represents the median of the data plotted left. Red line is the resulting curve from the regression analysis. Table shows the coefficients from the ordinary least squares (OLS). Statistical significance values represent the t-test against the null-hypothesis that the coefficient is zero. (c) Regression to a linear function does not fit the data as well as a quadratic function, which increases and decreases. Same experimental data are replotted here for comparison purposes. Left: Overlay of scatter plot with the linear function from the OLS. Right: Overlay of medians plotted with the linear function. Table shows the coefficients from the OLS.
(TIF)

Figure S7 **Overlay of** **Figure 4C and 4D** **onto the arena.** (a) In the absence of any food patch, fly position histogram shows a peak roughly 15–20 mm from the edge of the arena. (b) Comparison of 30 mm diameter of food patch (orange) to no food patch (blue, same data from Figure S7a replotted for comparison) and random distribution (teal). There is a clear difference in the distribution of fly positions between the arenas with the food patch vs. no food patch. The random distribution, expected if flies uniformly occupied the arena shows that it is qualitatively similar to no-food condition but very different from the arena with a 30 mm food patch.
(TIF)

Figure S8 **Activity in Gr5a^+^ GRNs is necessary for food-promoted aggression but not sufficient for normal levels of aggression.** (a) Inhibition of Gr5a^+^ GRNs by expression of UAS-TNT does not affect the level of aggression in the presence of females. n = 26, 32, 32 from left to right. Schematic figure shows the assay performed with a freeze-killed virgin female presented in the middle of the arena, partially embedded in agarose to prevent copulation. Two male flies are scored for aggressive behavior. (b) Inhibition of Gr5a^+^ GRNs by expression of UAS-Hid decreases sucrose-response (left, n = 4 and 4 for both genotypes. Each replicate has 10 male flies to calculate fraction of responders) and aggression in the presence of uniform food (right,n = 40 and 40 male-male pairs for both genotypes). (c) Silencing of Gr5a^+^ GRNs by expression of UAS-Shi^ts^ decreases aggression on 100 mM sucrose (n>26 for all conditions). d) Activation of Gr5a^+^ GRNs by expression of UAS-TRPV1 and UAS-NaChBac, tub-Gal80ts fails to increase aggression in the absence of food. n = 8, 12, 6, 8, 31, 34 for UAS-TRPV1 and 21, 31, 18, 35, 21, 49 for UAS-NaChBac Gal80ts. (e) Sucrose patch increases aggression to a level comparable to a food patch. n>84 for all three conditions tested.
(TIF)

Table S1 (XLSX)

Table S2 (XLSX)

Table S3 (XLSX)

Table S4 (XLSX)

Table S5 (XLSX)

## References

[1] Wang L, Han X, Mehren J, Hiroi M, Billeter J-C, et al.. (2011) Hierarchical chemosensory regulation of male-male social interactions in Drosophila. Nat Neurosci. doi:10.1038/nn.2800 10.1038/nn.2800 PMC3102769 21516101

[2] Wang L, Anderson DJ (2010) Identification of an aggression-promoting pheromone and its receptor neurons in Drosophila. Nature 463: 227–231 10.1038/nature08678 19966787 PMC2999963

[3] Chen S, Lee AY, Bowens NM, Huber R, Kravitz EA (2002) Fighting fruit flies: a model system for the study of aggression. Proc Natl Acad Sci USA 99: 5664–5668 10.1073/pnas.082102599 11960020 PMC122828

[4] Fernández M de LP, Chan Y-B, Yew JY, Billeter J-C, Dreisewerd K, et al (2010) Pheromonal and behavioral cues trigger male-to-female aggression in Drosophila. PLoS Biol 8: e1000541 10.1371/journal.pbio.1000541 21124886 PMC2990703

[5] Kurtovic A, Widmer A, Dickson BJ (2007) A single class of olfactory neurons mediates behavioural responses to a Drosophila sex pheromone. Nature 446: 542–546 10.1038/nature05672 17392786

[6] Jacobs M (1960) Influence of light on mating of Drosophila melanogaster. Ecology 41: 182–188.

[7] Nilsen SP, Chan Y-B, Huber R, Kravitz EA (2004) Gender-selective patterns of aggressive behavior in Drosophila melanogaster. Proc Natl Acad Sci USA 101: 12342–12347 10.1073/pnas.0404693101 15302936 PMC514477

[8] Vrontou E, Nilsen SP, Demir E, Kravitz EA, Dickson BJ (2006) fruitless regulates aggression and dominance in Drosophila. Nat Neurosci 9: 1469–1471 10.1038/nn1809 17115036

[9] Asahina K, Watanabe K, Duistermars BJ, Hoopfer E, González CR, et al (2014) Tachykinin-expressing neurons control male-specific aggressive arousal in Drosophila. Cell 156: 221–235 10.1016/j.cell.2013.11.045 24439378 PMC3978814

[10] Lu B, LaMora A, Sun Y, Welsh MJ, Ben-Shahar Y (2012) ppk23-Dependent chemosensory functions contribute to courtship behavior in Drosophila melanogaster. PLoS Genetics 8: e1002587 Available: http://eutils.ncbi.nlm.nih.gov/entrez/eutils/elink.fcgi?dbfrom=pubmed&id=22438833&retmode=ref&cmd=prlinks. 22438833 10.1371/journal.pgen.1002587 PMC3305452

[11] Pikielny CW (2012) Sexy DEG/ENaC channels involved in gustatory detection of fruit fly pheromones. Sci Signal 5: pe48 10.1126/scisignal.2003555 23131844

[12] Toda H, Zhao X, Dickson BJ (2012) The Drosophila Female Aphrodisiac Pheromone Activates ppk23(+) Sensory Neurons to Elicit Male Courtship Behavior. Cell Rep 1: 599–607 10.1016/j.celrep.2012.05.007 22813735

[13] Starostina E, Liu T, Vijayan V, Zheng Z, Siwicki KK, et al (2012) A Drosophila DEG/ENaC Subunit Functions Specifically in Gustatory Neurons Required for Male Courtship Behavior. J Neurosci 32: 4665–4674 Available: http://www.jneurosci.org/content/32/13/4665.full. 22457513 10.1523/JNEUROSCI.6178-11.2012 PMC3324785

[14] Thistle R, Cameron P, Ghorayshi A, Dennison L, Scott K (2012) Contact Chemoreceptors Mediate Male-Male Repulsion and Male-Female Attraction during Drosophila Courtship. Cell 149: 1140–1151 10.1016/j.cell.2012.03.045 22632976 PMC3365544

[15] Vijayan V, Thistle R, Liu T, Starostina E, Pikielny CW (2014) Drosophila pheromone-sensing neurons expressing the ppk25 ion channel subunit stimulate male courtship and female receptivity. PLoS Genetics 10: e1004238 10.1371/journal.pgen.1004238 24675786 PMC3967927

[16] Fernández MP, Kravitz EA (2013) Aggression and courtship in Drosophila: pheromonal communication and sex recognition. J Comp Physiol A Neuroethol Sens Neural Behav Physiol. doi:10.1007/s00359-013-0851-5 10.1007/s00359-013-0851-5 PMC3821735 24043358

[17] Billeter J-C, Levine JD (2012) Who is he and what is he to you? Recognition in Drosophila melanogaster. Curr Opin Neurobiol. doi:10.1016/j.conb.2012.08.009 10.1016/j.conb.2012.08.009 23010098

[18] Fan P, Manoli DS, Ahmed OM, Chen Y, Agarwal N, et al (2013) Genetic and neural mechanisms that inhibit Drosophila from mating with other species. Cell 154: 89–102 10.1016/j.cell.2013.06.008 23810192 PMC3823234

[19] Billeter J-C, Atallah J, Krupp JJ, Millar JG, Levine JD (2009) Specialized cells tag sexual and species identity in Drosophila melanogaster. Nature 461: 987–991 10.1038/nature08495 19829381

[20] Lacaille F, Hiroi M, Twele R, Inoshita T, Umemoto D, et al (2007) An inhibitory sex pheromone tastes bitter for Drosophila males. PLoS ONE 2: e661 10.1371/journal.pone.0000661 17710124 PMC1937024

[21] Chyb S, Dahanukar A, Wickens A, Carlson JR (2003) Drosophila Gr5a encodes a taste receptor tuned to trehalose. Proc Natl Acad Sci USA 100 Suppl 2 14526–14530 10.1073/pnas.2135339100 14523229 PMC304113

[22] Hoffmann AA, Cacoyianni Z (1990) Territoriality in Drosophila-Melanogaster as a Conditional Strategy. Animal Behaviour 40: 526–537.

[23] Yuan Q, Song Y, Yang C-H, Jan LY, Jan Y-N (2014) Female contact modulates male aggression via a sexually dimorphic GABAergic circuit in Drosophila. Nat Neurosci 17: 81–88 10.1038/nn.3581 24241395 PMC3995170

[24] Wang Z, Singhvi A, Kong P, Scott K (2004) Taste representations in the Drosophila brain. Cell 117: 981–991 10.1016/j.cell.2004.06.011 15210117

[25] Dahanukar A, Foster K, van der Goes van Naters WM, Carlson JR (2001) A Gr receptor is required for response to the sugar trehalose in taste neurons of Drosophila. Nat Neurosci 4: 1182–1186 10.1038/nn765 11704765

[26] Hoyer SC, Eckart A, Herrel A, Zars T, Fischer SA, et al (2008) Octopamine in male aggression of Drosophila. Curr Biol 18: 159–167 10.1016/j.cub.2007.12.052 18249112

[27] Skrzipek K, Kröner B (1979) Aggression bei Drosophila melanogaster— Laboruntersuchungen. Zeitschrift für ….

[28] Inagaki HK, Ben-Tabou de-Leon S, Wong AM, Jagadish S, Ishimoto H, et al (2012) Visualizing Neuromodulation In Vivo: TANGO-Mapping of Dopamine Signaling Reveals Appetite Control of Sugar Sensing. Cell 148: 583–595 Available: http://www.cell.com/abstract/S0092-8674(12)00009-8. 22304923 10.1016/j.cell.2011.12.022 PMC3295637

[29] Dethier VG (1976) The hungry fly: A physiological study of the behavior associated with feeding.

[30] Svetec N, Cobb M, Ferveur J-F (2005) Chemical stimuli induce courtship dominance in Drosophila. Curr Biol 15: R790–R792 10.1016/j.cub.2005.09.034 16213806

[31] Dahanukar A, Lei Y-T, Kwon JY, Carlson JR (2007) Two Gr genes underlie sugar reception in Drosophila. Neuron 56: 503–516 10.1016/j.neuron.2007.10.024 17988633 PMC2096712

[32] Slone J, Daniels J, Amrein H (2007) Sugar Receptors in Drosophila. Current Biology 17: 1809–1816 10.1016/j.cub.2007.09.027 17919910 PMC2078200

[33] USDA National Nutrient Database for Standard Reference, Release 24 (2011) USDA National Nutrient Database for Standard Reference, Release 24. U.S. Department of Agriculture, Agricultural Research Service. Available: http://www.ars.usda.gov/nutrientdata.

[34] Sweeney ST, Broadie K, Keane J, Niemann H, O'Kane CJ (1995) Targeted expression of tetanus toxin light chain in Drosophila specifically eliminates synaptic transmission and causes behavioral defects. Neuron 14: 341–351. 7857643 10.1016/0896-6273(95)90290-2

[35] Dow MA, Schilcher von F (1975) Aggression and mating success in Drosophila melanogaster. Nature 254: 511–512. 804664 10.1038/254511a0

[36] Keene AC, Masek P (2012) Optogenetic induction of aversive taste memory. Neuroscience 222: 173–180 10.1016/j.neuroscience.2012.07.028 22820051 PMC4006090

[37] Marella S, Fischler W, Kong P, Asgarian S, Rueckert E, et al (2006) Imaging taste responses in the fly brain reveals a functional map of taste category and behavior. Neuron 49: 285–295 10.1016/j.neuron.2005.11.037 16423701

[38] Gordon MD, Scott K (2009) Motor Control in a Drosophila Taste Circuit. Neuron 61: 373–384 10.1016/j.neuron.2008.12.033 19217375 PMC2650400

[39] Inagaki HK, Jung Y, Hoopfer ED, Wong AM, Mishra N, et al.. (2013) Optogenetic control of Drosophila using a red-shifted channelrhodopsin reveals experience-dependent influences on courtship. Nat Methods. doi:10.1038/nmeth.2765 10.1038/nmeth.2765 PMC4151318 24363022

[40] Kang K, Panzano VC, Chang EC, Ni L, Dainis AM, et al.. (2011) Modulation of TRPA1 thermal sensitivity enables sensory discrimination in Drosophila. Nature. doi:10.1038/nature10715 10.1038/nature10715 PMC3272886 22139422

[41] Harris TR (2010) Animal Behaviour: Multiple resource values and fighting ability measures influence intergroup conflict in guerezas (Colobus guereza). Animal Behaviour 79: 89–98.

[42] Zhou L, Schnitzler A, Agapite J, Schwartz LM, Steller H, et al (1997) Cooperative functions of the reaper and head involution defective genes in the programmed cell death of Drosophila central nervous system midline cells. Proc Natl Acad Sci USA 94: 5131–5136. 9144202 10.1073/pnas.94.10.5131 PMC24643

[43] Gray SJ, Jensen SP, Hurst JL (2002) Effects of resource distribution on activity and territory defence in house mice, Mus domesticus. Animal Behaviour 63: 531–539 10.1006/anbe.2001.1932

[44] Zhang YV, Raghuwanshi RP, Shen WL, Montell C (2013) Food experience-induced taste desensitization modulated by the Drosophila TRPL channel. Nat Neurosci 16: 1468–1476 10.1038/nn.3513 24013593 PMC3785572

[45] Dankert H, Wang L, Hoopfer ED, Anderson DJ, Perona P (2009) Automated monitoring and analysis of social behavior in Drosophila. Nat Methods 6: 297–303 10.1038/nmeth.1310 19270697 PMC2679418

[46] Manzo A (n.d.). In: Scott K, editor. Motor Neurons Controlling Fluid Ingestion in Drosophila melanogaster. UC Berkeley. 10.1073/pnas.1120305109 PMC3341050 22474379

[47] Armstrong DP (1991) Aggressiveness of Breeding Territorial Honeyeaters Corresponds to Seasonal Changes in Nectar Availability. Behavioral Ecology and Sociobiology 29: 103–111.

[48] Santangelo N, Itzkowitz M, Richter M (2002) Resource attractiveness of the male beaugregory damselfish and his decision to court or defend. Behavioral Ecology.

[49] Cummins SF, Boal JG, Buresch KC, Kuanpradit C, Sobhon P, et al (2011) Extreme aggression in male squid induced by a β-MSP-like pheromone. Curr Biol 21: 322–327 10.1016/j.cub.2011.01.038 21315594

[50] Rypstra AL, Schlosser AM, Sutton PL, Persons MH (2009) Multimodal signalling: the relative importance of chemical and visual cues from females to the behaviour of male wolf spiders (Lycosidae). Animal Behaviour 77: 937–947 10.1016/j.anbehav.2008.12.026

[51] Holldobler B, Lumsden CJ (1980) Territorial Strategies in Ants. Science 210: 732–739 10.1126/science.210.4471.732 17739532

[52] Mundiyanapurath S, Certel S, Kravitz EA (2007) Studying aggression in Drosophila (fruit flies). J Vis Exp: 155. doi:10.3791/155 10.3791/155 PMC2532939 18830427

[53] Guerra PA, Mason AC (2005) Information on Resource Quality Mediates Aggression between Male Madagascar Hissing Cockroaches, Gromphadorhina portentosa (Dictyoptera: Blaberidae). Ethology 111: 626–637 10.1111/j.1439-0310.2005.01086.x

[54] Root CM, Ko KI, Jafari A, Wang JW (2011) Presynaptic facilitation by neuropeptide signaling mediates odor-driven food search. Cell 145: 133–144 10.1016/j.cell.2011.02.008 21458672 PMC3073827

[55] Ueda A, Kidokoro Y (2002) Aggressive behaviours of female Drosophila melanogaster are influenced by their social experience and food resources. Physiol Entomol 27: 21–28 10.1046/j.1365-3032.2002.00262.x

[56] Watanabe K, Toba G, Koganezawa M, Yamamoto D (2011) Gr39a, a Highly Diversified Gustatory Receptor in Drosophila, has a Role in Sexual Behavior. Behav Genet. doi:10.1007/s10519-011-9461-6 10.1007/s10519-011-9461-6 21416142

[57] Toda H, Zhao X (2012) The Drosophila Female Aphrodisiac Pheromone Activates ppk23+ Sensory Neurons to Elicit Male Courtship Behavior. Cell Rep. 10.1016/j.celrep.2012.05.007 22813735

[58] van Swinderen B, Andretic R (2003) Arousal in Drosophila. Behav Processes. 10.1016/s0376-6357(03)00131-1 14556948

[59] Nitz DA, van Swinderen B, Tononi G, Greenspan RJ (2002) Electrophysiological correlates of rest and activity in Drosophila melanogaster. Curr Biol 12: 1934–1940. 12445387 10.1016/s0960-9822(02)01300-3

[60] Wang L, Dankert H, Perona P, Anderson DJ (2008) A common genetic target for environmental and heritable influences on aggressiveness in Drosophila. Proc Natl Acad Sci USA 105: 5657–5663 10.1073/pnas.0801327105 18408154 PMC2311352

[61] Certel SJ, Savella MG, Schlegel DCF, Kravitz EA (2007) Modulation of Drosophila male behavioral choice. Proc Natl Acad Sci USA 104: 4706–4711 10.1073/pnas.0700328104 17360588 PMC1810337

[62] ANTONY C, JALLON J (1982) The chemical basis for sex recognition in Drosophila melanogaster. J Insect Physiol 28: 873–880 10.1016/0022-1910(82)90101-9

[63] Cobb M, Jallon J-M (1990) Pheromones, mate recognition and courtship stimulation in the Drosophila melanogaster species sub-group. Animal Behaviour 39: 1058–1067 10.1016/S0003-3472(05)80778-X

[64] Smith JM, Price GR (1973) The Logic of Animal Conflict. Nature.

[65] Hixon MA, Carpenter FL, Paton DC (1983) Territory Area, Flower Density, and Time Budgeting in Hummingbirds: An Experimental and Theoretical Analysis. Am Nat.

[66] Adams E (2001) Approaches to the study of territory size and shape. Annual Review of Ecology and Systematics.

[67] Martin J-R (2004) A portrait of locomotor behaviour in Drosophila determined by a video-tracking paradigm. Behav Processes 67: 207–219 10.1016/j.beproc.2004.04.003 15240058

[68] Simon JC, Simon JC, Dickinson MH, Dickinson MH (2010) A New Chamber for Studying the Behavior of Drosophila. PLoS ONE 5: e8793 10.1371/journal.pone.0008793.g007 20111707 PMC2811731

[69] Leopold LF, Leopold N, Diehl H-A, Socaciu C (2011) Quantification of carbohydrates in fruit juices using FTIR spectroscopy and multivariate analysis. Journal of Spectroscopy 26: 93–104 10.3233/SPE-2011-0529

[70] Lewis EB (1960) Lewis: A new standard food medium - Google Scholar. Drosophila Information Service.

[71] Cornmeal, Sucrose, Dextrose, Yeast and 2-Acid Medium (n.d.) Cornmeal, Sucrose, Dextrose, Yeast and 2-Acid Medium. Bloomington Drosophila Stock Center. Available: http://flystocks.bio.indiana.edu/Fly_Work/media-recipes/caltechfood.htm. Accessed 2014 May 16.

[72] Hoffmann AA (1987) A Laboratory Study of Male Territoriality in the Sibling Species Drosophila-Melanogaster and Drosophila-Simulans. Animal Behaviour 35: 807–818.

[73] Schwartz NU, Zhong L, Bellemer A, Tracey WD (2012) Egg laying decisions in Drosophila are consistent with foraging costs of larval progeny. PLoS ONE 7: e37910 10.1371/journal.pone.0037910 22693584 PMC3365076

[74] Grosjean Y, Rytz R, Farine J-P, Abuin L, Cortot J, et al.. (2011) An olfactory receptor for food-derived odours promotes male courtship in Drosophila. Nature. doi:10.1038/nature10428 10.1038/nature10428 21964331

[75] Lone SR, Sharma VK (2012) Or47b receptor neurons mediate sociosexual interactions in the fruit fly Drosophila melanogaster. J Biol Rhythms 27: 107–116 10.1177/0748730411434384 22476771

[76] Pfeiffer BD, Truman JW, Rubin GM (2012) Using translational enhancers to increase transgene expression in Drosophila. Proc Natl Acad Sci USA 109: 6626–6631 10.1073/pnas.1204520109 22493255 PMC3340069

