# Supplementary figures and images for: How Food Controls Aggression in Drosophila
Source: PLoS One. 2014 Aug 27;9(8):e105626. doi: 10.1371/journal.pone.0105626 (PMC4146546; doi:10.1371/journal.pone.0105626)

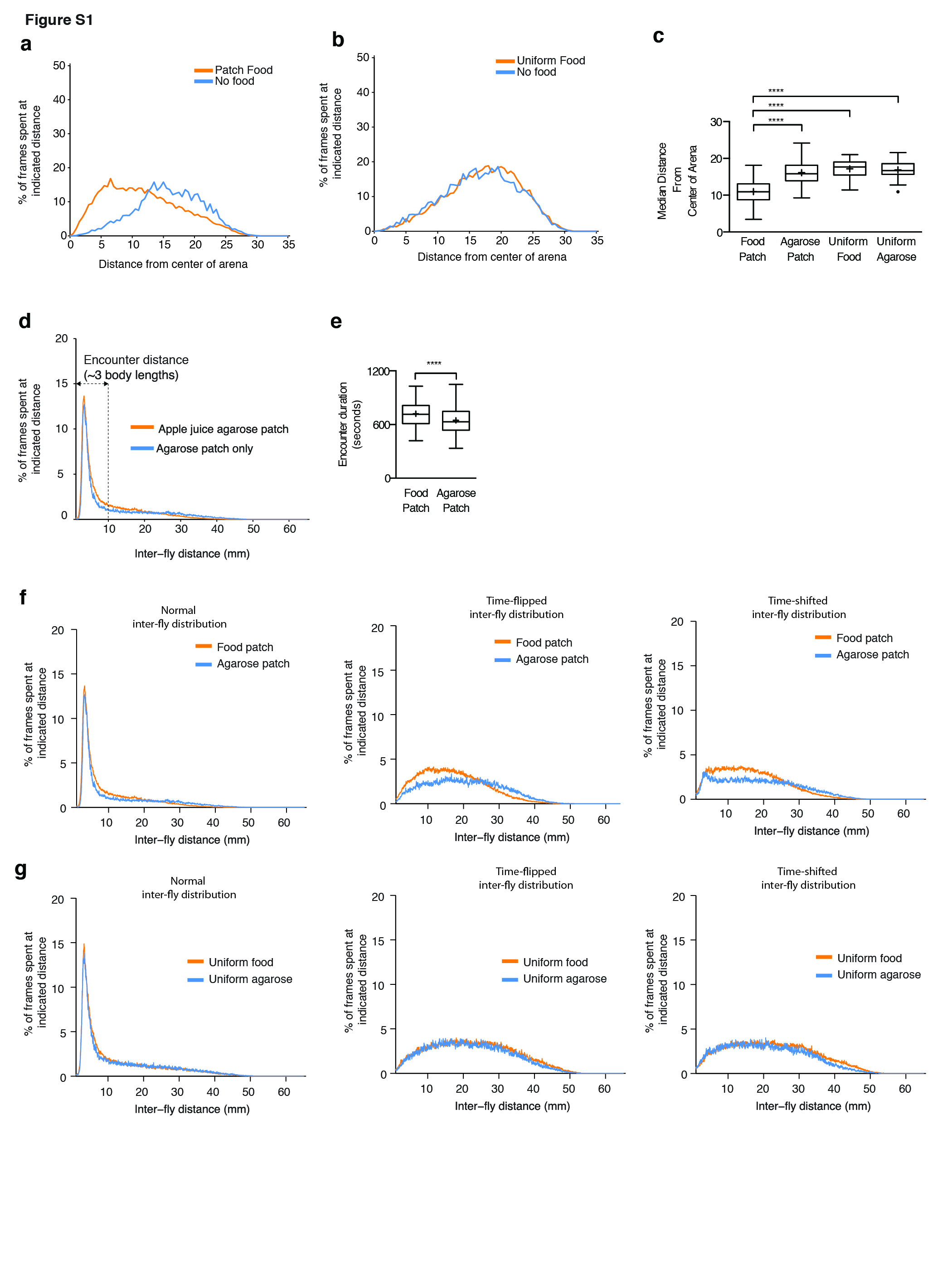

Supplement: Figure S1 — Proximity between two male flies is changed by the presence of a small food patch but not by uniform food. (a) In the presence of a small food patch, there is clear attraction to the center of the arena. n = 171 and 92 for food patch and agarose patch, respectively. n = 72 and 44 for uniform food and uniform agarose, respectively. The pairs are further analyzed for all of Supplemental Figures 1. (b) In the presence of food, which covers the surface of the arena uniformly, there is no change in the distribution of the flies with respect to the center of the arena. (c) Quantification of the data in (a) and (b): Median distances from the center of the arena are changed in the presence of a small food patch. (d) Inter-fly distance histogram shows that the presence of a small food patch slightly changes the distribution compared to the absence of food. (e) Sum of the encounter (inter-fly distance <10 mm) duration shows that the presence of a small patch of food slightly increases the amount of time flies spend within 10 mm of each other. (f) Left: Same data as (d) replotted for comparison. Middle: Shows the same data as Left after transformation of the position of one fly with respect to time by flipping the order (first frame becomes last frame and vice versa). Transformation shows that flies are naturally attracted to the center of the arena but the prominent encounter peak is not present, suggesting that the peak depends on the coordinated positioning of two flies. Right: Shows the results of similar transformation as Middle but instead of flipping the order, 1000 frames were added to shift one fly's position with respect to time. (g) Left: Same data as Figure 1D replotted for comparison. Middle and Right: Transformation as performed in (f) shows that the presence of uniform food does not change the position of flies and that the prominent peak in inter-fly distance histogram is likely due to the natural interaction distance of flies. (TIF) [file pone.0105626.s001.tif]

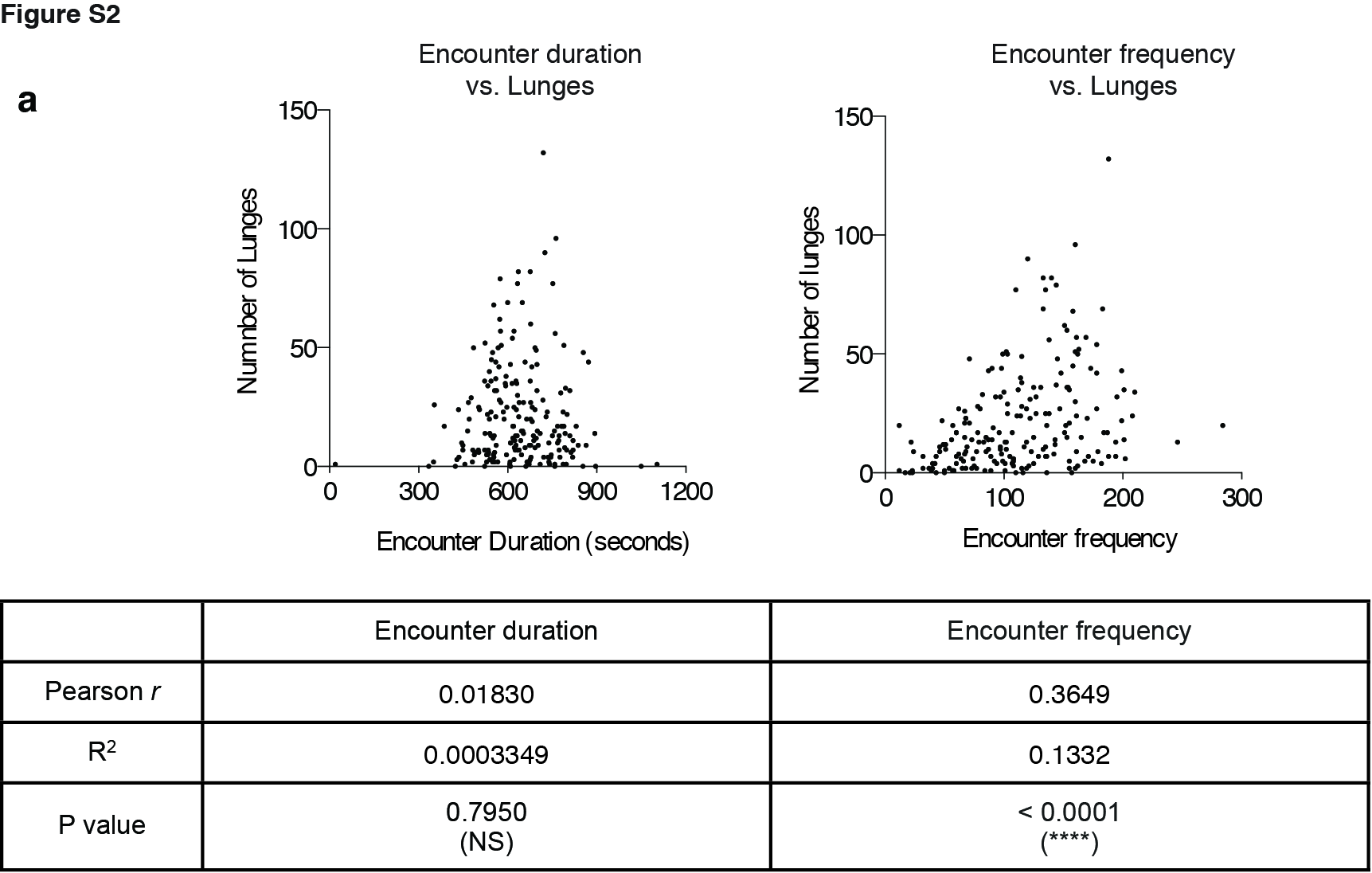

Supplement: Figure S2 — Encounter duration is an independent measure of aggression. (a) Encounter duration, the amount of time flies spend within 10 mm of each other, shows no correlation (r = 0.018) with the number of lunges. Most of the points lie near the 600 seconds (50% of the assay) regardless of the number of lunges observed. n = 204 x, y pairs. (b) Encounter frequency, the number of times flies come within 10 mm of each other, shows a weak correlation (r = 0.365) with the number of lunges. (TIF) [file pone.0105626.s002.tif]

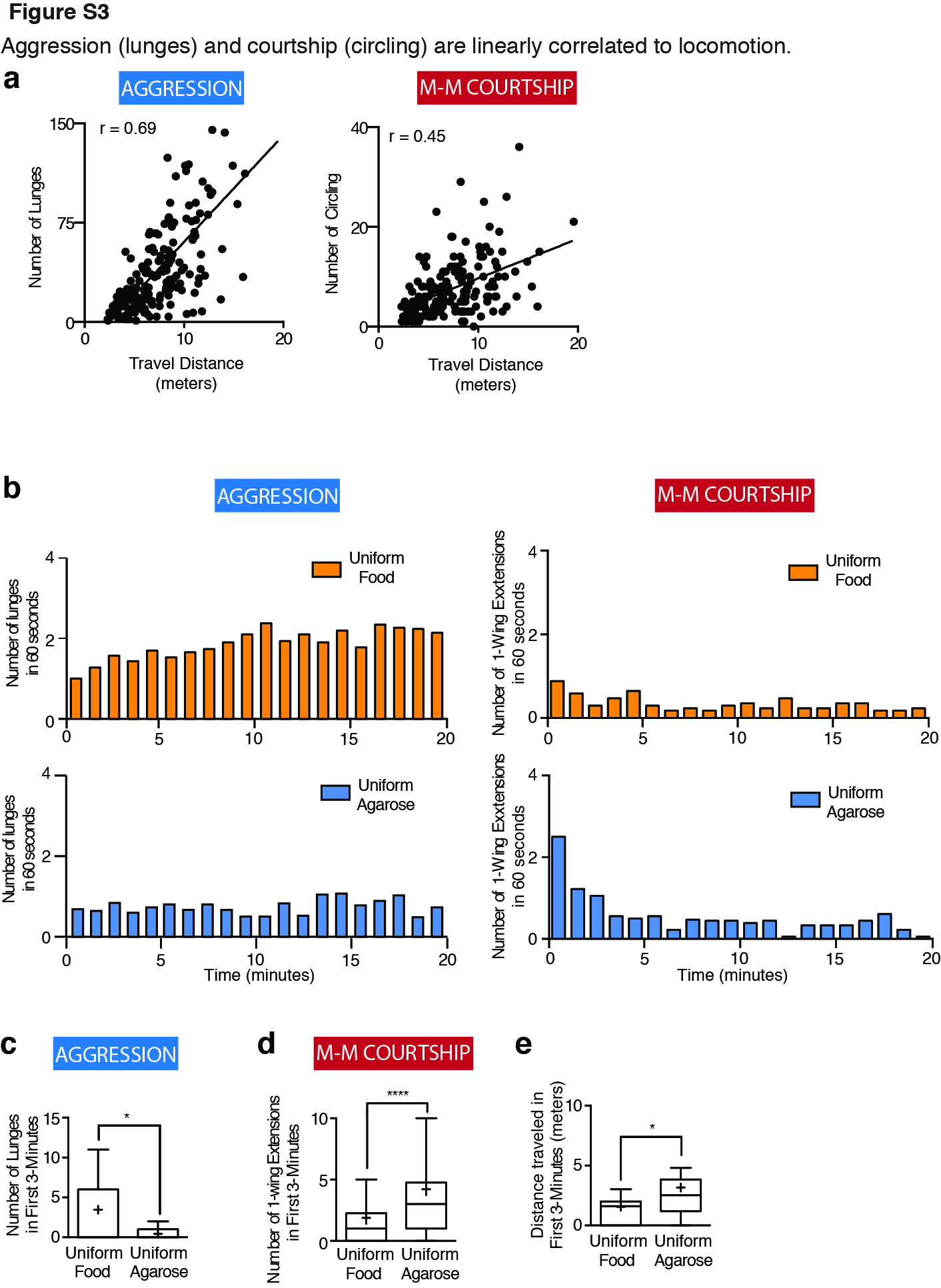

Supplement: Figure S3 — Food promotes aggression and not courtship. (a) Left: Aggression (number of lunges, y-axis) is linearly correlated with locomotion (r = 0.69, travel distance in meters on x-axis). Right: Courtship (number of circling) is linearly correlated with locomotion (r = 0.45). n = 171 male-male pairs. (b). Behavioral choice between male-male courtship and male-male aggression develops in the first three minutes of the assay and remains stable. Left: Aggression increases slightly over time in the presence of food (orange). No change is observed in the absence of food (blue). n = 113 for uniform food and 44 for uniform agarose. Right: Male-male courtship (one-wing extension) decreases slightly over time in the presence of food (orange) and without food (blue). One-wing extension data were manually scored. n = 18 and 17 for uniform food and agarose, respectively. (c) Presence of food increases aggression in the first three minutes of the assay. Manually scored lunges for male-male pairs, n = 33 and 33 for food and agarose conditions. (d) Presence of food decreases male-male courtship (one-wing extensions) in the first three minutes of the assay. Manually scored one-wing extensions for male-male pairs, n = 34 and 31 for food and agarose conditions for one-wing extensions. (e) Presence of food decreases locomotion in the first three minutes of the assay. n = 34 and 31 for food and agarose. (TIF) [file pone.0105626.s003.tif]

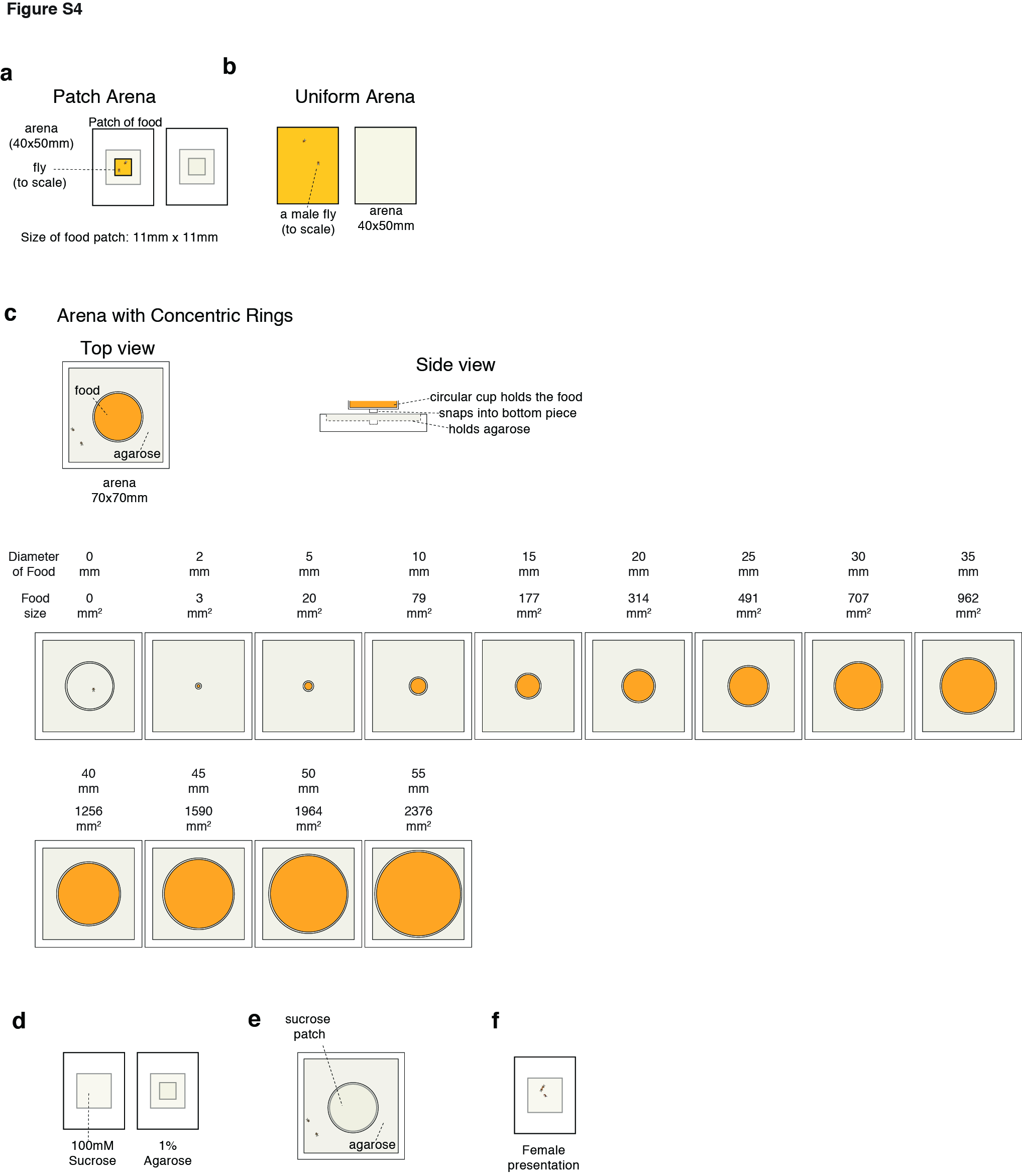

Supplement: Figure S4 — Schematic diagrams of all of the arenas used in behavioral assays. (a) Patch arena: 11 mm×11 mm food patch is used and compared with agarose. Surrounding the food patch there is an area with agarose. The arena is 40 mm×50 mm. (b) The uniform arena has the entire surface covered with either food or agarose. (c) An arena with concentric rings allows for testing of multiple sizes of food with diameters. The food patch is surrounded by agarose, which is surrounded by a small plastic base. The entire arena is 70 mm×70 mm. (d) Experiments with the sucrose patch were performed with either sucrose or agarose in a 22 mm×22 mm square area in the middle of the arena. (e) Experiments testing different sucrose concentrations (0, 100, 200, 800 mM) were performed with 707 mm2 patch of sucrose. (f) Experiments testing female-induced aggression were performed with 40 mm×50 mm arena with a dead female on top of an agarose patch in the middle. (TIF) [file pone.0105626.s004.tif]

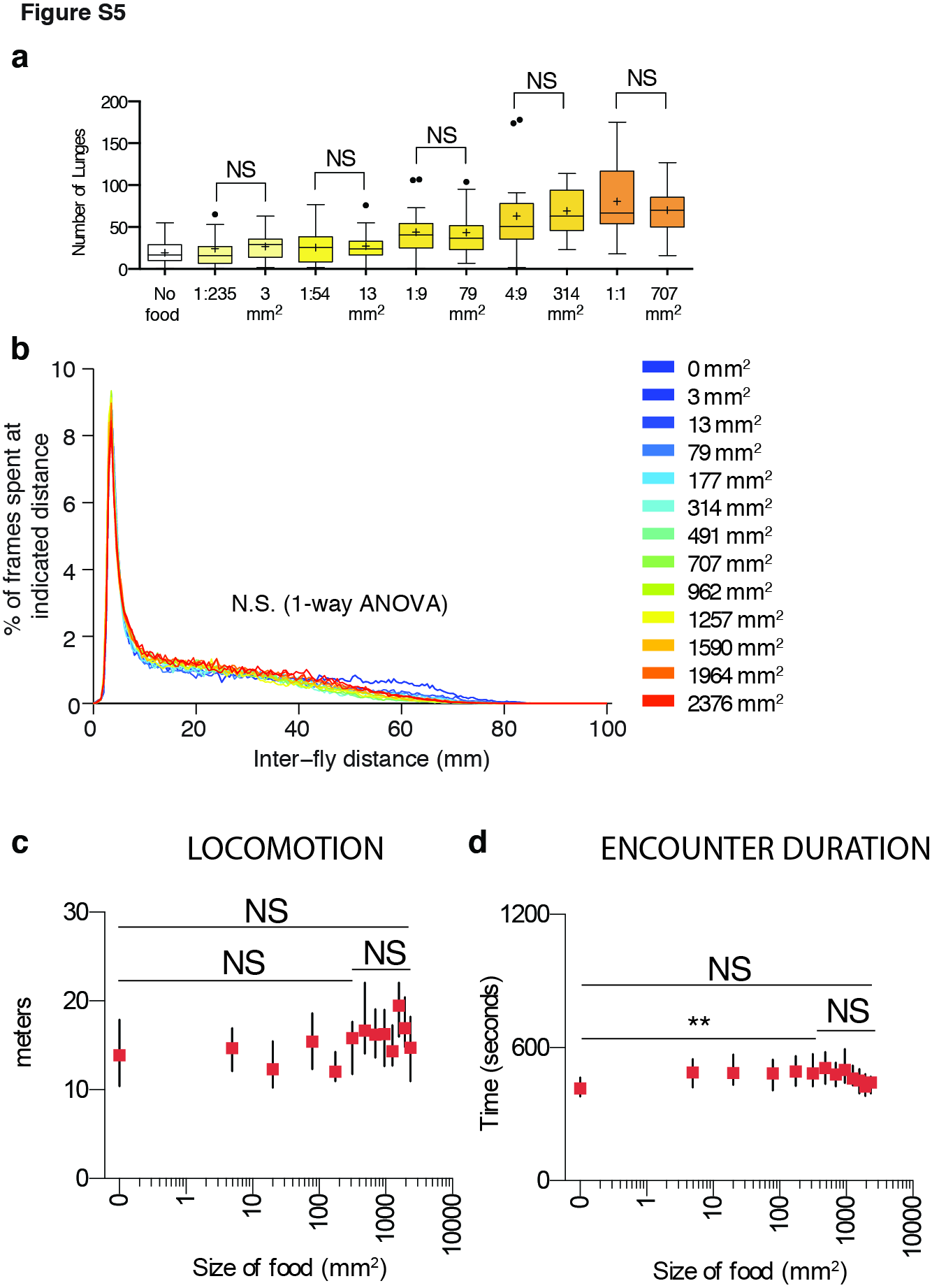

Supplement: Figure S5 — (a) The absolute amount of food, rather than concentration or area of food, determines the level of aggression (1∶235 dilution of food with 707 mm2 area is equivalent to a 3 mm2 food patch, etc). Every dilution–size pair is statistically indistinguishable from the other condition. The data are replotted from Figure 2e for comparisons. (b) Inter-fly distribution shows the pattern of inter-fly distance does not change over 13 different sizes of food patch ranging from 0 to 2376 mm2 does not change the pattern of inter-fly distance (1-way ANOVA). n>28 for all conditions. (c) Locomotion shows little to no change as the size of food changes from 0 to 2376 mm2. See Table S2 for details. n>28 for all conditions. (d) Encounter duration shows no change as the size of food changes from 0 to 2376 mm2. See Table S5 for statistics. n>28 for all conditions. (TIF) [file pone.0105626.s005.tif]

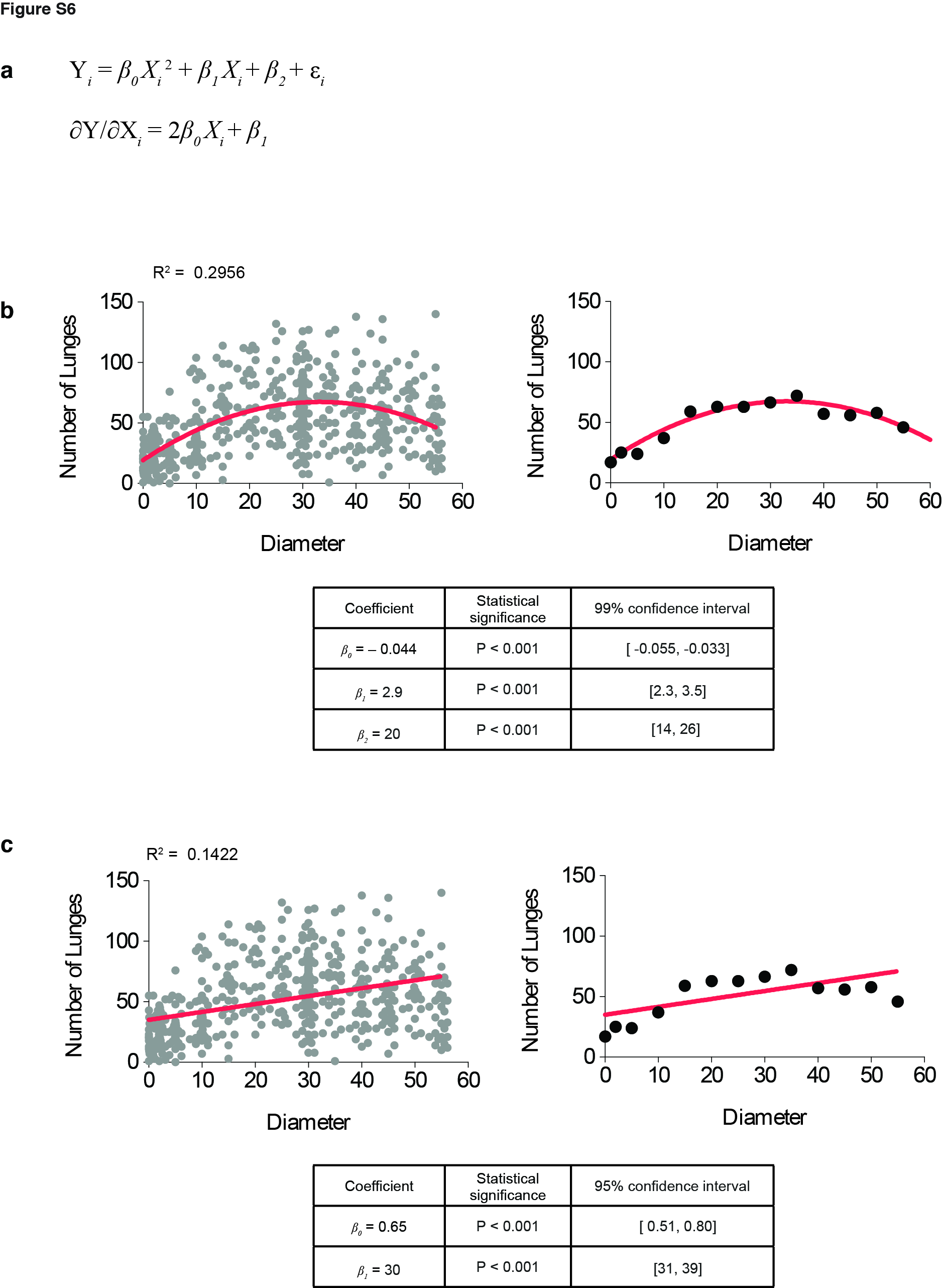

Supplement: Figure S6 — Aggression shows biphasic response to the amount of food. (a) Functional form being tested for curve-fitting analysis. (b) Curve-fitting the quadratic function of the form in (a) shows that there is an increasing and decreasing pattern. Left: Scatter plot of the experimental data (n = 493). x-axis is diameter of food and y-axis is number of lunges. Right: Each dot represents the median of the data plotted left. Red line is the resulting curve from the regression analysis. Table shows the coefficients from the ordinary least squares (OLS). Statistical significance values represent the t-test against the null-hypothesis that the coefficient is zero. (c) Regression to a linear function does not fit the data as well as a quadratic function, which increases and decreases. Same experimental data are replotted here for comparison purposes. Left: Overlay of scatter plot with the linear function from the OLS. Right: Overlay of medians plotted with the linear function. Table shows the coefficients from the OLS. (TIF) [file pone.0105626.s006.tif]

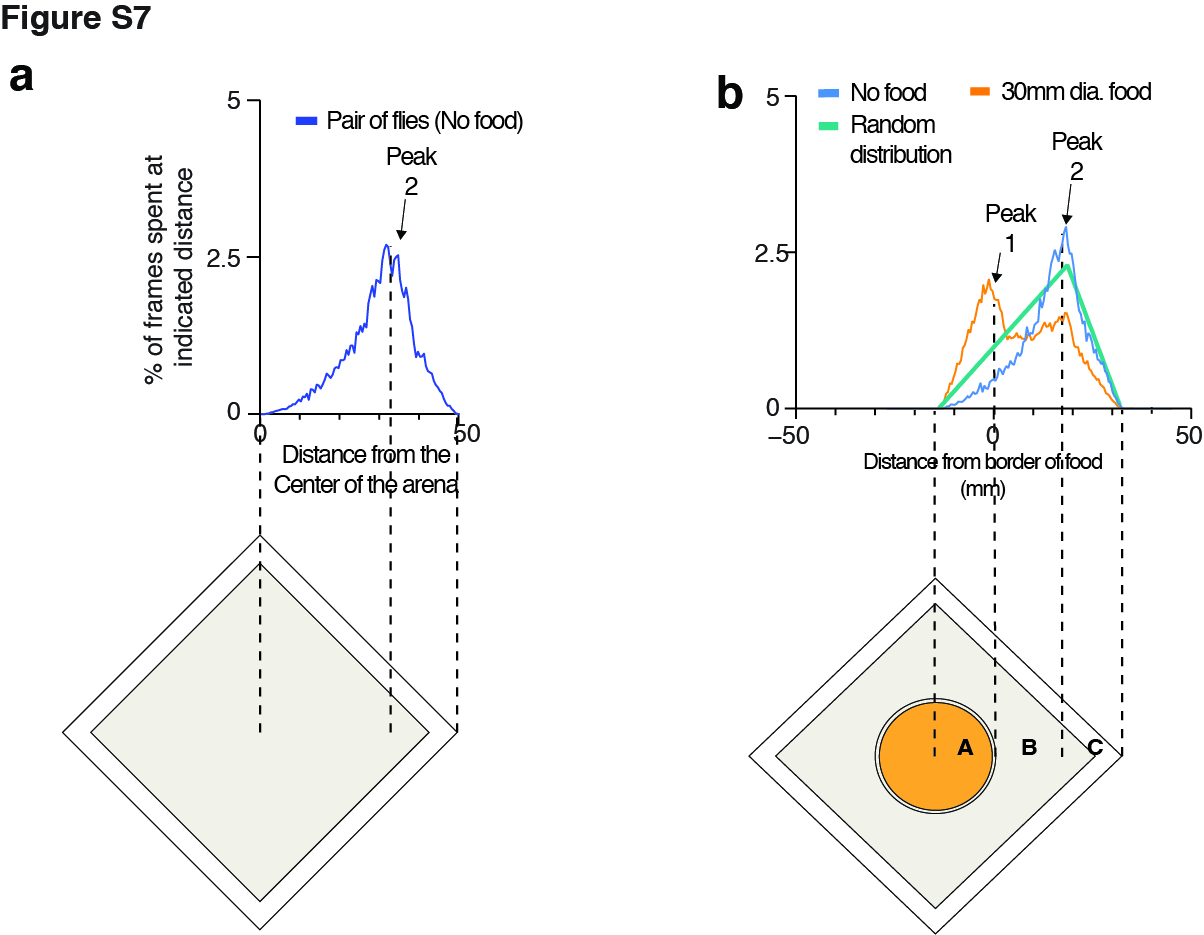

Supplement: Figure S7 — Overlay of Figure 4C and 4D onto the arena. (a) In the absence of any food patch, fly position histogram shows a peak roughly 15–20 mm from the edge of the arena. (b) Comparison of 30 mm diameter of food patch (orange) to no food patch (blue, same data from Figure S7a replotted for comparison) and random distribution (teal). There is a clear difference in the distribution of fly positions between the arenas with the food patch vs. no food patch. The random distribution, expected if flies uniformly occupied the arena shows that it is qualitatively similar to no-food condition but very different from the arena with a 30 mm food patch. (TIF) [file pone.0105626.s007.tif]

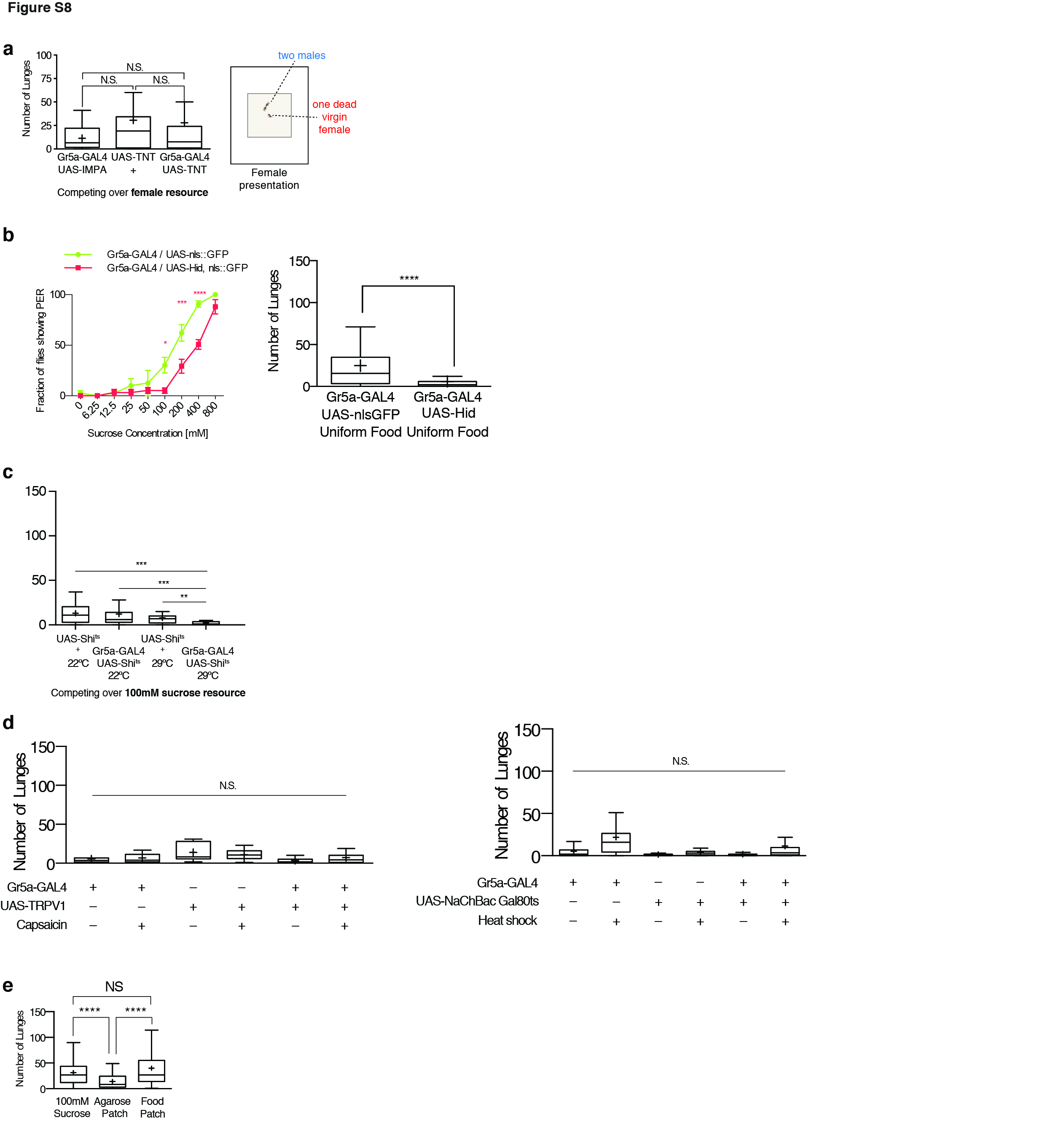

Supplement: Figure S8 — Activity in Gr5a+ GRNs is necessary for food-promoted aggression but not sufficient for normal levels of aggression. (a) Inhibition of Gr5a+ GRNs by expression of UAS-TNT does not affect the level of aggression in the presence of females. n = 26, 32, 32 from left to right. Schematic figure shows the assay performed with a freeze-killed virgin female presented in the middle of the arena, partially embedded in agarose to prevent copulation. Two male flies are scored for aggressive behavior. (b) Inhibition of Gr5a+ GRNs by expression of UAS-Hid decreases sucrose-response (left, n = 4 and 4 for both genotypes. Each replicate has 10 male flies to calculate fraction of responders) and aggression in the presence of uniform food (right,n = 40 and 40 male-male pairs for both genotypes). (c) Silencing of Gr5a+ GRNs by expression of UAS-Shits decreases aggression on 100 mM sucrose (n>26 for all conditions). d) Activation of Gr5a+ GRNs by expression of UAS-TRPV1 and UAS-NaChBac, tub-Gal80ts fails to increase aggression in the absence of food. n = 8, 12, 6, 8, 31, 34 for UAS-TRPV1 and 21, 31, 18, 35, 21, 49 for UAS-NaChBac Gal80ts. (e) Sucrose patch increases aggression to a level comparable to a food patch. n>84 for all three conditions tested. (TIF) [file pone.0105626.s008.tif]
